# Glass Transition, Crystallization of Glass-Forming Melts, and Entropy

**DOI:** 10.3390/e20020103

**Published:** 2018-02-01

**Authors:** Jürn W. P. Schmelzer, Timur V. Tropin

**Affiliations:** 1 Albert-Einstein-Strasse 23-25, 18059 Rostock, Germany; 2 Frank Laboratory of Neutron Physics, Joint Institute for Nuclear Research, Joliot-Curie 6, 141980 Dubna, Russia

**Keywords:** crystal nucleation, entropy, Kauzmann paradox, 64.60.Bd General theory of phase transitions, 64.60.Q- Nucleation, 4.70.D- Solid–liquid transitions, 64.70.kj Glasses, 64.70.Q- Theory and modeling of the glass transition, 65.40.gd Entropy

## Abstract

A critical analysis of possible (including some newly proposed) definitions of the vitreous state and the glass transition is performed and an overview of kinetic criteria of vitrification is presented. On the basis of these results, recent controversial discussions on the possible values of the residual entropy of glasses are reviewed. Our conclusion is that the treatment of vitrification as a process of continuously breaking ergodicity with entropy loss and a residual entropy tending to zero in the limit of zero absolute temperature is in disagreement with the absolute majority of experimental and theoretical investigations of this process and the nature of the vitreous state. This conclusion is illustrated by model computations. In addition to the main conclusion derived from these computations, they are employed as a test for several suggestions concerning the behavior of thermodynamic coefficients in the glass transition range. Further, a brief review is given on possible ways of resolving the Kauzmann paradox and its implications with respect to the validity of the third law of thermodynamics. It is shown that neither in its primary formulations nor in its consequences does the Kauzmann paradox result in contradictions with any basic laws of nature. Such contradictions are excluded by either crystallization (not associated with a pseudospinodal as suggested by Kauzmann) or a conventional (and not an ideal) glass transition. Some further so far widely unexplored directions of research on the interplay between crystallization and glass transition are anticipated, in which entropy may play—beyond the topics widely discussed and reviewed here—a major role.

## 1. Introduction

As noted long ago by Josiah Willard Gibbs [1], “*Any method involving the notion of entropy, the very existence of which depends on the second law of thermodynamics, will doubtless seem to many far-fetched, and may repel beginners as obscure and difficult of comprehension*”. Similarly, John von Neumann mentioned that “*nobody knows what entropy really is, and if you use the word ‘entropy’ in an argument, you will win every time*”.

As clearly indicated by these statements, the use of the concept of entropy for different applications, such as the analysis of matter in thermodynamic equilibrium [2,3] or of fluctuations [4] and evolution processes in relatively different systems [5], including the understanding of the “arrow of time” [6], is not only characterized by enormous successes but also by the necessity to cope with and to overcome highly non-trivial problems. Such problems have continued to be discussed until now. Some of these that we have been partly engaged consist in the proper definition of entropy, including applications beyond thermodynamics and statistical physics [7,8,9]; the compatibility of the “arrow of time” with classical and quantum mechanics [10,11]; the definition of temperature for systems of finite size, including the question of whether temperature fluctuations exist or not [12,13]; and the question of how the properties of critical clusters, determining the rate of nucleation, have to be correctly specified [14].

Problems with the entropy concept and its correct interpretation evolved also in the discussion of the third law of thermodynamics formulated first by Walther Nernst [15] (see also [16,17]). For example, Albert Einstein in 1914 [18] tried to give an interpretation of the third law of thermodynamics (Nernst’s theorem, as it was also denoted at that time) on the basis of quantum theory concepts. He then concluded that Nernst’s theorem is valid for one-component crystalline solids but not for mixtures. The applicability of Nernst’s theorem to glasses he left open, then motivating it by the argument that the nature of the glassy state is still not understood.

These topics have been discussed subsequently by a variety of outstanding scientists and have been reviewed in detail by Franz Simon in 1937 in [19]. Simon noted there that if it would be “*postulated that Nernst’s theorem should be applied only to pure crystals …this would be a very severe restriction, so severe, in fact, that Nernst’s theorem could no longer be regarded as a general law at all …Summing up, we can state that the present experimental evidence indicates the general validity of Nernst’s theorem as a law of thermodynamics. The possibility that some future experiment may not be in agreement with the theorem obviously cannot be excluded, but unless there is some reason from a theoretical point of view to expect such a result, to anticipate it is mere speculation.*” In this discussion, he notes as well that the situation is quite different for frozen-in systems, that is, glasses.

Different formulations of the third law of thermodynamics can be found also in the monographs by Max Planck, for example, in [20,21]. Already in the third edition of his book [21] in 1910 he noted that his interpretation goes beyond the original formulation by Nernst, leaving open the possibility to return to Nernst’s treatment provided his extension turns out not to be correct. Also with respect to the general formulation of the third law of thermodynamics, extending it to multicomponent systems in thermodynamic equilibrium as expressed, for example, in the monograph [21] of Planck, different opinions can be found in the literature. For example, Bazarov [22] denies that the third law has a satisfactory statistical–mechanical interpretation, while Landau & Lifshitz [3] state the opposite—that the third law can be given a foundation only in terms of quantum statistics. In any case, with respect to systems in thermodynamic equilibrium, the situation became well settled in the course of the discussions, partly confirming the proud words of Nernst, that “*with the third principle of thermodynamics the whole development of thermodynamics has come to its natural end: nothing of significance should follow*” [23]. This statement is to some extent correct with respect to equilibrium thermodynamics; however, it ignores the subsequent extension of classical thermodynamics to non-equilibrium states closely connected with the work of de Donder, Prigogine and Defay, Mandelstam, Leontovich, and others (see, e.g., [24,25] for an overview).

In addition, as already noted in connection with Einstein’s remarks, one of the major problems of discussion was whether glasses obey the third law of thermodynamics or not (see, e.g., [26,27,28,29,30,31,32]). For example, Simon [30,31,32] analyzed in detail the behavior of the entropy of the liquid in glass formation in comparison with the entropy of the crystalline phase. Considering first helium as a singular substance that can be cooled down to temperatures near to absolute zero remaining in a metastable equilibrium state, he demonstrated that the entropy difference, Δs, of liquid and crystal tends to zero in this limit. As he noted further, this must be the case in accordance with the third law of thermodynamics valid in its classical formulation for equilibrium states of the systems under consideration. The commonly observed different type of behavior of liquids he described by considering, as he noted, glycerol as an example.

As he explained in detail, in cooling, starting at the melting temperature, the entropy difference between the metastable liquid and the crystalline phase decreases and becomes nearly constant once the liquid has been transformed into a glass. He explained then that such behavior is not in conflict with the third law of thermodynamics clearly expressing that glasses as non-equilibrium systems do not obey the third law of thermodynamics. Simon did not formulate or implicitly indicate the existence of any “*first entropy crisis*” as stated by Wolynes [33] referring to Simon’s paper [32]. Instead, Simon noted that, except helium, liquids do not exist in equilibrium in the vicinity of absolute zero; by this reason, the third law is not applicable to them. Simon also already discussed the possibility to formulate the third law in a form valid both for systems in thermodynamic equilibrium as well as for glasses in the form of the principle of unattainability of the absolute zero of temperature [30]. The advantages and limitations of this more general formulation as compared to Planck’s formulation of the third law of thermodynamics are analyzed in detail in [16,34,35].

A similar behavior as discussed by Simon for the vitrification of glass-forming melts had been observed later also for crystals, for which disorder can be generated in different ways (e.g., point defects, impurities, dislocations, etc.) and can become frozen-in in cooling [36,37]. Particular examples of this type of behavior have been treated by Linus Pauling in [38], but he was not at all the first to give a correct interpretation of the entropy of glasses or glass-like systems at low temperatures (cf. [33]). In such a way, Simon also resolved in advance some more recently arising problems connected with claims of the necessity to reconsider the behavior of entropy in vitrification [24,35,36,39]. This problem is briefly reviewed and illustrated here on the basis of the methods of thermodynamics of irreversible processes and a simple but sufficiently accurate statistical–mechanical model of glass-forming melts, proving the correctness of the classical interpretation of Simon and others (Section 3). As a preliminary step and starting point of this analysis, we briefly discuss the basic definition of glass and the glass transition, including recent proposals to reformulate it (Section 2). These results are extended in Section 4 in a review of the Kauzmann paradox and its implications. A summary of results and further perspectives (Section 5) completes the paper.

## 2. Glass and the Glass Transition

### 2.1. Basic Definitions and Some Comments

As described in detail in [24], a first definition of glasses was proposed by Gustav Tammann (see, e.g., his monograph [40]), denoting it as undercooled solidified melts. This definition was considerably expanded by Simon [30,31,32], who suggested to consider glasses as kinetically frozen-in thermodynamically non-equilibrium systems, distinguishing glasses from amorphous systems in thermodynamic equilibrium. Hereby it is assumed in a first approximation—suggested also by Simon—that the transformation takes place at some well-defined discrete temperature, the glass transition temperature, $T_{g}$. Simon was aware, of course, that the glass transition proceeds not at a discrete temperature value but over a certain temperature range. He had already also noted with reference to Tammann and Kohlhaas [41] and Parks and Huffmann [42] explicitly that by varying the cooling rate, different glasses can be obtained. However, he considered both the width of this glass transition range and the effect of varying cooling rates on glass properties as small and, for this reason, to be of minor importance [32]. Such reservations are today known to be not adequate, particularly if wide ranges of cooling and heating rates are employed while available [43].

Since these first fundamental considerations by Tammann and Simon, a variety of different approaches has been advanced in order to understand the detailed mechanism of the glass transition and, in its connection, the nature of the vitreous state (e.g., [44,45,46,47,48]; an overview is given in [49]). However, despite the different treatments, when posing the question as to which of the principal states of matter (solid, liquid, or gas; see, e.g., [50]) glass belongs to, in line with the classical treatment of Simon and Tammann, glasses have to be considered as a solid. Glasses behave as a solid in the absolute majority of applications. Indeed, as noted in a frequently cited statement by the Nobel laureate P. W. Anderson, “*The deepest and most interesting unsolved problem in solid state theory is probably the theory of the nature of glass and the glass transition*” [51].

Already by this reason we consider it as unreasonable to treat glasses as “*a state of matter that appears solid on a short time scale but continuously relaxes towards the liquid state*” as supposed in an advanced recent modification of the definition of glass by Zanotto and Mauro [52]. The latter property—to change its state in time scales “*which exceed the limits of human history*” (see discussion below)—is not a specific feature of glasses as was well-known already by Heraclitus. His “*pantha rhei*” or “*everything flows*” refers not only to glasses. Crystals, rivers, mountains (see the subsequent discussion of the Deborah number), and so forth also flow on sufficiently large time scales. Because everything flows on such historical time scales, this feature is not a specific property of glasses and cannot be used to distinguish it from any other states of matter.

The fact that predominantly glasses flow with a perceptible rate on relevant time scales only in a certain temperature range and not beyond is well known in glass technology [24], as is clearly formulated also for example by Tammann [40,41] and is already given in the title of his well-known paper by Tool [53]. Of course, for certain applications, flow processes have to be taken into consideration as is well known already from the work of R. & F. Kohlrausch, Weber, Williams & Watts, Adams & Williamson, Eyring & Tobolsky (see, e.g., [54]) starting around 1850. However, such possible flow processes under certain conditions have not been considered as essential by Tammann and Simon in their definition of glass. Such a point of view, that flow processes may be neglected in most applications for relevant times scales, has also been clearly expressed by one of the authors of [52] in [55,56]. For example, in [55] it is noted that “*window glasses may flow at ambient temperature only over incredibly long times, which exceed the limits of human history*”. The flow processes considered in [55,56] are primarily the response to external fields and are governed by viscosity. However, the viscosity and structural relaxation time are uniquely correlated as noted also in [55,56], where the analysis is performed widely in terms of relaxation times.

In [52], Zanotto and Mauro discuss Simon’s definition of glass as a freezing-in process. Referring to [30] and presenting Simon’s point of view in the form that “*glass is a rigid material obtained from freezing-in a supercooled liquid in a narrow temperature range*”, they further state that “*it is not clear if he intended to convey the same meaning we are using here (frozen = a temporary state)*”. However, the meaning Simon assigned to his statement of freezing-in is clearly reflected in [32]. In free translation, it sounds as though freezing-in at $T_{g}$ does not imply that below $T_{g}$ relaxation processes are excluded. However, any such structural transformations proceed already slightly below $T_{g}$ with such large time scales that the suggestion of a permanent arrest of such structural changes is completely substantiated ([32], p. 223); or, as stated by Davies and Jones ([57], p. 375), “*Simon pointed out that as a glass is cooled through its transformation temperature the molecular diffusion which is necessary to effect the appropriate change in configuration is increasingly inhibited and finally becomes practically impossible*”. This interpretation is fully in line with [55,56] but not with the revised definition of a glass given in [52].

Moreover, the viscosity of glass-forming melts increases dramatically with decreasing temperature [24,58]. One of the relations describing it with a sufficiently high degree of accuracy for most applications is the Vogel–Fulcher–Tammann equation widely employed in glass science [59]. This equation results in a divergence of the viscosity at finite values of temperature, denoted as Vogel temperatures. Whether the viscosity will really diverge or not is a matter of intensive debate; it cannot be established by direct experimental investigations restricted to maximum values of viscosity less than $10^{18}$ Pa·s. In any case, a variety of models of the vitreous state lead to the confirmation of such a conclusion. However once the viscosity diverges, the structural relaxation time also diverges. Glasses at temperatures below the Vogel temperature are then excluded from the vitreous state by the above-mentioned definition.

Further extending their modification of the definition of glass, Zanotto and Mauro propose to include into the definition of glass the statement, “*Their ultimate fate, in the limit of infinite time, is to crystallize*”. However, even if this statement would be true, it seems to us not to be reasonable to include such a statement into the definition, as it does not supply any additional information as to what glasses are. In addition, if at all, crystallization proceeds at a perceptible rate for states below the glass transition range also only at time scales exceeding the limits of human history.

**Figure 1:**
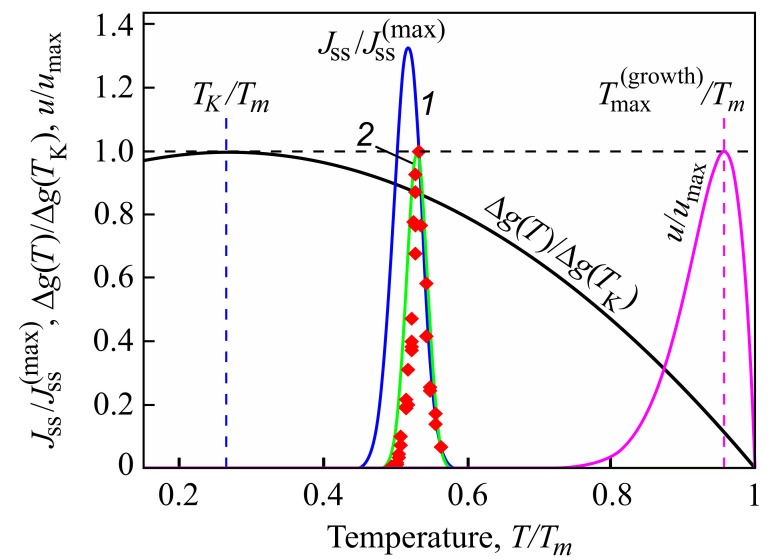
Normalized steady-state nucleation rate, $J_{ss}/J_{ss}^{\left(max\right)}$, and normalized crystal growth rate, $u/u_{max}$, in dependence on reduced temperature, $T/T_{m}$. Here $J_{ss}^{\left(max\right)}$ is the maximum nucleation rate and $u_{max}$ is the maximum growth rate obtained via experiment; $T_{m}$ is the melting or liquidus temperature. The blue curve (1) shows the theoretical result when the kinetic term in the expression for the nucleation rate is determined via appropriate diffusion coefficients; the green curve (2) is drawn under the assumption of validity of the Stokes–Einstein–Eyring equation, allowing one to replace the diffusion coefficient with viscosity. Its wide coincidence with experimental data is reached by employing appropriate expressions for the curvature dependence of the surface tension (for details, see [64]). The reduced thermodynamic driving force, $\Delta g\left(T\right)/\Delta g\left(T_{K}\right)$, is also shown; it has a maximum at the Kauzmann temperature, $T_{K}$ [65]. It is evident that crystallization occurs only in a relatively small temperature range. Typically the maximum of the growth rate, $T_{max}^{\left(growth\right)}$, is located at temperatures much higher than the maximum of the steady-state nucleation nucleation rate [60], as shown here in the figure.

Indeed, from a theoretical point of view, it can be shown that intensive nucleation occurs in a relatively small range of temperatures (see Figure 1), with a maximum near to the glass transition temperature defined in the classical form as related to a viscosity of $10^{12}$ Pa·s [40]. This maximum is caused by the interplay of thermodynamic and kinetic factors dominating the crystal nucleation process [60,61]. The kinetic factor is correlated with viscosity (more precisely, with diffusion coefficients, but qualitatively this leads to the same results [62]), significantly increasing with decreasing temperature. As a consequence, perceptible nucleation does not occur for a variety of glasses under normal conditions on earth. They are completely excluded in cases when the viscosity approaches infinity (or the appropriate diffusion coefficients become equal to zero). Even if local nucleation processes do proceed, this does not result necessarily in complete crystallization, as the maximum of the growth rates is located, as a rule, at much higher temperatures [60]. For this reason, in experiments, Tammann’s development method [24,61,63] is commonly employed in the analysis of crystal nucleation. The formation of crystal nuclei is stimulated by choosing some well-defined nucleation temperature; then in order to detect these nuclei, the temperature is switched to higher values to allow them to grow to detectable experimental sizes over reasonable experimental time scales.

As one consequence, in [52], obsidian and amber are mentioned as particular examples of natural glasses. However, the oldest samples of amber known are $320\times 10^{6}$ years old and have not crystallized until now [66]. According to [67], “*obsidian is found in many locations worldwide. It is confined to areas of geologically recent volcanic activity. Obsidian older than a few million years is rare because the glassy rock is rapidly destroyed or altered by weathering, heat, or other processes*”. Consequently, “*it flows*”, but not into the direction of the state of a crystal. There is no indication by geologists that obsidian may be transformed in the course of time into crystals. In addition, in the course of human history, people have employed these glasses for different purposes and have invented new glasses without having to cope with the problem of their crystallization at normal exploitation conditions [24,68,69]. Strong support of the point of view that glasses do not crystallize at normal conditions in relevant time scales can be found also in [70]. In this paper, this fact is even distinguished as the “*paradox of old glasses*”, although it can be understood straightforwardly by the theoretical considerations sketched above. Summarizing, glasses may crystallize under certain conditions, but this is not the general rule in their evolution.

The definition of a glass as advanced by Tammann and Simon has some limitations; it is strongly connected with the traditional method of producing glasses via cooling. It can be easily adapted to glass transition and glass fabrication via variation of pressure or other similar external control parameters. In any such cases, the glass is formed via a glass transition, the transformation of a (metastable) equilibrium liquid into a frozen-in non-equilibrium state, the glass. Once it is formed, glass can no longer “*exhibit a glass transition*”, as stated several times, and even in the advanced modified definition of glass in [52], (“*glass is a non-equilibrium, non-crystalline condensed state of matter that exhibits a glass transition*”).

Once one attempts to propose a modern definition of glass as formulated as one of the aims of mentioned paper, one should also, at least, discuss other types of glasses, such as disordered crystals [36,37,38], and alternative well-known and frequently discussed methods of their preparation, such as vapor or electrolytic deposition processes [24,70]. Of course, it is not so easy to adapt the standard definition of glasses by Simon to such cases. Attempts to advance a definition of glass that is valid independently of the means of its preparation can be found in the second edition of [24].

### 2.2. What Is the Right Deborah Number?

The notation “phase” was introduced into thermodynamics by Gibbs [71] exclusively for the description of systems in thermodynamic equilibrium (e.g., Gibbs’s phase rule specifies the number of thermodynamic degrees of freedom for equilibrium states). Consequently, glass is (by definition) not a thermodynamic phase. The glass transition from the metastable liquid to a glass and vice versa is already by this reason not an (equilibrium) phase transition. This statement is further confirmed by the fact that, instead of thermodynamic relations determining the states in which equilibrium phase transitions take place, pressure and temperature of vitrification and devitrification are determined essentially by kinetic factors such as, for example, the rate of change of temperature or pressure. For this reason, kinetic criteria have to be at one’s disposal to determine the glass transition temperature or pressure.

Following the pioneering work of Bartenev [72,73,74] and Ritland [75], it became widely accepted in the glass community that the glass transition, for example, in the cooling of a melt, occurs at a temperature $T_{g}$ specified by some ratio of characteristic time scales, one of these being the characteristic (Maxwellian) relaxation time, $\tau_{R}$, of the system under consideration [72,73,74,75] or its derivative with respect to temperature (Volkenstein & Ptizyn [76,77], Cooper & Gupta [78]). Hereby it is supposed that the Maxwellian time relation determines the rate of approach of the structure (or the structural-order parameters) to metastable equilibrium. In its simplest form, when one structural-order parameter, ξ, is sufficient for the description of the deviation of the system under consideration from equilibrium, the dependence of the structural order-parameter on time, *t*, can be described via a relation:

(1) $$\frac{d\xi }{dt}=-\frac{1}{\tau_{R}\left(p,T,\xi \right)}\left(\xi -\xi_{e}\right)$$

formulated and employed for the first time (as far as we are aware) by Bragg and Williams [24,79] for crystals with substitutional disorder and introduced into glass science somewhat later by Tool [53]. Here $\xi_{e}$ is the equilibrium value of the structural-order parameter identified by Tool with the fictive temperature. Once the dependence of the structural order-parameter on time is known, all other parameters of the system under consideration and their dependence on time can be established as well provided their dependence on the structural order-parameter is available from experiment or theoretical models. In general, the relaxation times of these other characteristics of the system will have different values as compared to the relaxation time entering Equation (1) since the respective quantities are as a rule non-linear functions of the structural order-parameter. In any case, the degree of evolution of the system to the actual equilibrium value of the structural order-parameter at fixed values of pressure and temperature is determined then by the ratio of observation time, the time the experiment is conducted, to the value of the relaxation time. Similar ideas are frequently also employed in the specification of the glass transition temperature. Here, however, some specific features have to be accounted for which are often overlooked as will be evident from the subsequent analysis.

Indeed, in the discussion of kinetic criteria for vitrification the Deborah number is quite frequently mentioned. As expressed, for example, recently in [52], it is stated that “*$T_{g}$ refers to a temperature where the time of observation (of an experiment), $t_{obs}$, is of the same order as the average structural relaxation time, $\tau_{R}$, of the supercooled liquid; $t_{obs}≅\tau_{R}$ … Hence, at $T_{g}$ the $\left(\tau_{R}/t_{obs}\right)$-ratio (the well-known Deborah number [80]) is unity*”.

However, several problems arise here: (i) The relaxation time is quantitatively specified in the paper, but the time of observation is not. Without such specification, the criterion does not give any definite predictions concerning the value of the glass transition temperature. (ii) The second problem is that kinetic criteria of glass formation have been formulated being not of the form as stated there. In particular, in [81], the Lillie number is discussed (employing the criterion advanced in [76,77,78]), being different as compared to the ratio mentioned in [52]. Consequently, the question arises as to what the basic kinetic criterion is and how the different kinetic criteria may be correlated. (iii) Third, the Deborah number is not relevant for the specification of the glass transition (see also [24,82,83,84]). It refers to the description of structural reorganization or annealing processes at given external control parameters but not to the response of the system to variations of external control parameters like pressure and temperature.

Indeed, Marcus Reiner distinguished in his short but influential paper [80] between liquids and solids and not between liquids and glasses. He noted, in particular, “*Heraclitus’s ‘everything flows’ was not entirely satisfactory. Were we to disregard the solid and deal with fluids only? The way out of this difficulty had been shown by the Prophetess Deborah even before Heraclitus. In her famous song after the victory over the Philistines, she sang, ‘The mountains flowed before the Lord’. When, over 360 years ago, the Bible was translated into English, the translators, who had never heard of Heraclitus, translated the passage as ‘The mountains melted before the Lord’ and so it stands in the authorized version. But Deborah knew two things. First, that the mountains flow, as everything flows. But, secondly, that they flowed before the Lord, and not before the man, for the simple reason that man in his short lifetime cannot see them flowing, while the time of observation of God is infinite. We may therefore well define as a non-dimensional number the Deborah number, Dh = (Time of relaxation)/(Time of observation). The difference between solids and fluids is then defined by the magnitude of Dh …*”. He then continued, “*In every problem of rheology make sure that you use the right Deborah number*.”

As shown in [82,83,84], the correct ratio of time scales (the “*right Deborah number*" in Reiner’s notation) determining the glass transition temperature, $T_{g}$, is not the ratio of observation and relaxation times but of the characteristic time of change of external control parameters and the relaxation time (see also [44]). This conclusion is demonstrated in [82,83,84] to be a direct consequence of basic thermodynamic considerations. For glass transition caused by cooling or heating, the characteristic time of change of temperature, $\tau_{T}$, is defined via a relation similar to Equation (1) as

(2) $$\frac{dT}{dt}=-\frac{1}{\tau_{T}}T$$

with $\tau_{R}≅\tau_{T}$; we obtain then a general kinetic criterion of glass transition of the form

(3) $$\tau_{R}≅\tau_{T}\;,\;{\left\{\frac{1}{T}\left|\frac{dT}{dt}\right|\tau_{R}\right\}}_{T=T_{g}}≅1$$

This criterion holds for both cooling and heating. The respective glass transition temperatures may hereby differ even for cooling and heating with the same absolute values of the rate of change of temperature since the relaxation time depends on the structural order-parameter exhibiting a hysteresis curve (see Section 3.2). We note that similar considerations can be employed also for the description of vitrification caused by variations of pressure or other appropriate thermodynamic control parameters.

As shown in [82,83,84], this criterion contains all particular kinetic criteria of glass formation derived up until now ($q\tau_{R}≅1$ [73,74,75], $q(d\tau_{R}/dT)≅1$ [76,77,78], where q=(dT/dt) is the rate of change of temperature) as (approximate) special cases (see also [85]), including the criterion for a dynamic glass transition ($\omega \tau_{R}≅1$, ω being the angular frequency of external perturbations; for the derivation, see [83]) first formulated by Bartenev [72]. It gives a more precise formulation of the criterion $q\tau_{R}≅1$ advanced first by Bartenev and Ritland and removes uncertainties connected with the possibility of different choices of the temperature scale to be employed in this relation. Moreover, a derivation of Equation (3) with respect to temperature immediately results in $q(d\tau_{R}/dT)≅1$ derived first by Volkenstein and Ptizyn. In addition, this approach allows one also to specify the conditions for glass formation in vapor or electrolytic deposition, as discussed in detail in [24]. Beyond this, a variety of additional consequences could be derived, including the determination of the width of the glass transition interval [86] and the specification of the pressure dependence of the glass transition temperature.

Indeed, taking the derivative of Equation (3) with respect to pressure, we obtain the following (neglecting variations of the structural-order parameters with pressure [83]; such effects can also be easily accounted for [84]):

(4) $$\frac{dT_{g}}{dp}=-{\left.\frac{{\left(\frac{\partial \tau_{R}}{\partial p}\right)}_{T}}{{\left(\frac{\partial \tau_{R}}{\partial T}\right)}_{p}-\frac{\tau_{R}}{T}}\right|}_{T=T_{g}}\;$$

It follows as a consequence that the pressure dependence of the glass transition temperature at fixed values of the rate of change of temperature is determined by the dependence of the relaxation time on pressure and temperature.

As one additional consequence, these results lead to relations for the pressure dependence of the glass transition temperature that are different from the predictions of Ehrenfest-type equations. Under certain conditions, one of them can be obtained as an approximate special case [83,87,88]. Because only one equation is obtained, moreover, no definite estimates concerning the value of the Prigogine–Defay ratio follow as a consequence. For this reason, any discussion concerning the problem of why its value deviates from that in glass transition becomes superfluous [83,87,88]. In the present paper, we employ Equation (3) for an analysis of the Kauzmann paradox as discussed in Section 4.

## 3. Residual Entropy of Glasses

### 3.1. A Brief Overview of Some Recent Discussions

In the present section, we give a brief review of some recent controversial discussions concerning the residual entropy of glasses that are supposed to have been finally resolved—as sketched in the introduction—in the course of the first half of the last century. The revival of the interest in these problems can be clearly correlated with the complexity of the notion of entropy and the ways of its determination mentioned already by Gibbs and von Neumann. Meanwhile, the interest in this topic has decreased. However, in view of the continuing and even growing interest in the correct theoretical description of the glass transition and the nature of the vitreous state, it seems to be quite useful to give an overview of its course and the results. Such an overview is also required as until now, some of the concepts proven to be incorrect have survived the discussion and may lead to incorrect results in applications, as demonstrated, for example, in [89]. This review is by necessity brief and incomplete, but the main directions are intended to be reflected.

The renewal of the discussions on the residual entropy of glasses can be traced back to two papers by Kivelson and Reiss [90,91]. The starting point of Kivelson and Reiss was the statement that “*metastable states are not in true equilibrium and so cannot be directly treated by thermodynamics and statistical thermodynamics*”. They proposed an alternative thermodynamic treatment to overcome these difficulties. As they noted, the resulting procedure “*permits one to treat metastable systems consistently within a completely time independent and causal thermodynamic framework. It also gives a consistent description of the entropy of glassy and similar random metastable systems in which the entropy vanishes at T→0 K, and it explains the apparent residual entropy at 0 K obtained in most conventional analyses based upon experiments carried out over irreversible paths (but not always recognized as such)*” [90]. About 10 years later, Howard Reiss added the following [91]: “*The importance of a thermodynamic framework is tied to the fact that evolving molecular theories of residual entropy, which also impact the glass transition, no matter how sophisticated, invariably contain approximations whose effects are hard to assess… It strongly suggests that residual entropy, in general, is an impression that stems from the inclusion of an irreversible step in an experimental thermodynamic cycle*”.

According to mentioned authors, thermodynamics and statistical physics are not directly applicable to metastable states. Indeed, in line with a well-known theorem of Yang and Lee [92], thermodynamic quantities must have a singularity at states of phase equilibrium. The appearance of metastable states in the statistical description is mainly a result of certain assumptions employed in the theory. In addition, homogeneous nucleation theory postulates the presence of a certain spectrum of heterophase fluctuations in a metastable phase. If this is the case, such an ensemble of fluctuations should contribute to the thermodynamic properties of the system under consideration. These effects can be expected to be small for states near to the binodal curve but may increase with increasing supersaturation and, in particular, near to the spinodal curve. Only the thermodynamic functions not accounting for heterophase fluctuations may be considered as analytic functions of their arguments (for more details, see [93]).

However, measurements of a variety of properties of liquids show no singularities in passing from stable to metastable states [94], and the majority of applications of nucleation theory to experiments involve the computation of the thermodynamic driving force and the specific interfacial energy for metastable systems based on thermodynamics extending results for the thermodynamic quantities obtained for equilibrium to non-equilibrium states. The results give in a variety of cases not only a qualitative but also a quantitatively correct description of the experimental data. Consequently, the question arises as to how grave the uncertainties truly are when thermodynamics is applied to metastable states. Once the modification of the thermodynamic description proposed by Kivelson and Reiss leads to such grave consequences, a similar question arises as posed by them in applications to statistical models: Is their approach really the only possible approach, and does it contain, perhaps, ingredients that are not appropriate? Does it really refer to glasses once it is stated that the theory “*permits one to treat metastable systems consistently within a completely time independent and causal thermodynamic framework*.” However, glasses are not metastable but thermodynamically non-equilibrium systems.

The second main statement in the cited part of the papers is that the assumption of the existence of residual entropy is an illusion resulting from the inclusion of irreversible processes in the thermodynamic methods of the determination of the entropy. In Section 3.2, we return to the analysis of this statement, modeling the glass transition in terms of the thermodynamics of irreversible processes in combination with statistical–mechanical models of liquids. In particular, we analyze the problem of the effect of entropy production on vitrification. This problem was first raised and studied long ago by Davies and Jones [57,95] and resulted in similar conclusions as will be derived here on the basis of the model computations: irreversible processes are of negligible importance for the determination of the residual entropy of glasses. This conclusion is reconfirmed also by other studies reviewed below.

The point of view of Kivelson and Reiss concerning the values of the residual entropy of glasses was adopted by Gupta, Mauro, and others, claiming [96] “*that: (i) there is an entropy loss associated with the liquid to glass transition, and (ii) the configurational entropy in the glassy state vanishes at absolute zero*”. In [97], they “*propose a generalized definition of entropy accounting for the continuous breakdown of ergodicity at the laboratory glass transition …The continuous loss of ergodicity during the laboratory glass transition is accompanied by a loss of entropy as the system gradually becomes trapped in a subset of the configurational phase space …In all cases, the entropy of glass is zero in the limit of absolute zero temperature, since here the system is necessarily confined to a single microstate*”. This point of view is further elaborated on in [98], where the following is stated: “*A common assumption in the glass science community is that the entropy of a glass can be calculated by integration of measured heat capacity curves through the glass transition. Such integration assumes that glass is an equilibrium material and that the glass transition is a reversible process. However, as a non-equilibrium and non-ergodic material, the equations from equilibrium thermodynamics are not directly applicable to the glassy state*”.

First it has to be noted here that glasses as frozen-in systems do obey the equations of equilibrium thermodynamics. These equations are consequences of the first and second laws of thermodynamics. Once relaxation processes are of negligible significance for frozen-in systems, entropy production terms can be completely neglected. Only in the glass transition range do they have to be accounted for. Employing this approach, a variety of thermodynamic properties of glasses can be predicted and analyzed as explored in detail in [24]. As discussed here in the introduction and further in the present section, peculiarities occur only with respect to the third law of thermodynamics. As is also evident from these considerations, different definitions of the vitreous state are not merely the expression of different points of view having no severe consequences; instead, they are of huge direct significance in glass science and its applications.

Moreover, in the cited papers, the ergodic hypothesis is introduced into the discussion. The introduction of such a concept does not, from our point of view, lead to any clarification of the problems under consideration. Indeed, as noted in [99], “*An … older controversy, which in the opinion of some physicists has long ceased to be an interesting problem, concerns the ergodic hypothesis … It states that the time-average value of an observable—which of course is determined by the dynamics—is equivalent to an ensemble average, that is, an average at one time over a large number of systems, all of which have identical thermodynamic properties but are not identical on the molecular level … The general consensus is that the hypothesis, still mathematically unproven, is probably true yet irrelevant for physics*” (see also [100]).

We do believe that this statement is completely correct, although other opinions can also be found in the literature [25,101], demonstrating at the same time the problems and uncertainties arising in the application of such concepts. Given the above-cited statement holds already for systems in thermodynamic equilibrium, what can be expected then from its application to systems considered at varying boundary conditions or concerning systems in frozen-in non-equilibrium states? In any case, the conclusion concerning the value of entropy is derived not from any consequences of a well-founded theory of continuously broken ergodicity, but from estimates of the number of accessible microstates a given system is trapped to in cooling. This number of microstates of the single system is considered then as the property determining the entropy. However, statistical physics always deals with averages over ensembles of identical systems and not with single systems. Mentioned considerations cannot be used, consequently, for a correct specification of the value of the entropy.

In detail, a critical analysis of sketched above point of view of Gupta and Mauro et al. has been performed, in particular, by Martin Goldstein [102,103,104] and has been shown to not be correct. In particular, in [102], Goldstein noted, “*We show that the hypothesis that the configurational entropy of a liquid disappears when it is kinetically frozen into a single glass state …implies directly the possibility of an uncompensated conversion of heat to work. We also note that the number of microstates visited in the course of a measurement does not determine the entropy, but rather that this number is always an inconceivably small fraction of the accessible microstates*”. In other words [103], “*the assumption that the configurational entropy of a supercooled liquid vanishes at $T_{g}$ leads to a non-trivial violation of the second law…The most parsimonious conclusion from these results is that residual entropies are real*”.

In [104], Goldstein advances his considerations on the correlation between entropy and the number of microstates visited: “*… the view has been expressed by some who hold the zero entropy view that, to measure entropy, all or an appreciable number of the microstates that contribute to the entropy must be visited. We show here that the entropy calculated on the basis of the number of microstates visited during any conceivable time of measurement would be underestimated by at least 20 orders of magnitude …We conclude that calorimetrically measured residual entropies are real*”.

In an alternative form concentrating on its consequences, Gyan P. Johari discussed this problem. He noted in [105], “*A postulate that ergodicity and entropy continuously decrease to zero on cooling a liquid to a glassy state was used to support the view that glass has no residual entropy, and the features of mechanical relaxation spectra were cited as proof for the decrease. We investigate whether such spectra and the relaxation isochrones can serve as the proof. We find that an increase in the real component of elastic moduli with an increase in spectral frequency does not indicate continuous loss of ergodicity and entropy, and the spectra do not confirm isothermal glass transition or loss of entropy*”. In [106], he added, “*In support of the view that entropy is lost on glass formation, the $C_{p}^{\prime }$ relaxation spectra were regarded as experimental proof of time- and temperature-dependent loss of both ergodicity and entropy, and confirmation of isothermal glass transition. Also, both $C_{p}\to 0$ and S→0 in the limits of $t_{obs}\to 0$ s, and T→0 K were cited as further proof … The notions of partial ergodicity and entropy and their dependence on $t_{obs}$ are inconsistent with the properties measured during cooling, heating and isothermal annealing, and thermodynamic consequences of the apparent proof are untenable. The premise that glass formation is a process of continuously breaking ergodicity with entropy loss does not merit serious consideration*”.

Additional support of the traditional point of view on the existence of residual entropy of glasses was given by a variety of authors by very different argumentations. Richet [107] showed, in particular, that “*the traditional view that glasses possess residual entropy, which can be determined by calorimetric means, is quantitatively supported by applications of Adam and Gibbs configurational entropy theory to the temperature, composition and pressure dependencies of the viscosity of silicate melts. This theory is also in harmony with the mechanisms of viscous flow, as understood from nuclear magnetic resonance experiments …*”. Conradt [108] and Fotheringham et al. [109] discussed entropy differences between frozen-in states and the equilibrium states for a variety of systems, “*showing that the conventional entropy of a frozen-in phase at zero Kelvin assumes a non-zero residual value …*”. They discussed also the effect of entropy production and evaluated this as negligible, confirming the results of the previously mentioned analysis by Davies and Jones [57,95]. Support of the traditional point of view on the residual entropy of glasses is also given by a recent analysis performed by Nemilov [110]. In this respect, it is worth noting that Nemilov discussed already long ago in [25] broken-ergodicity concepts and their relevance for the understanding of glasses. He provides also an overview there on a variety of problems and attempts of their resolution in the application of this concept. Most importantly, in connection with the discussion performed here, he as well as the authors of the papers referenced by him did not come to any conclusions conflicting with the existence of a residual entropy of glasses; in contrast, the broken-ergodicity concept is utilized in the cited reference in support of this experimentally well-established result. Claims that broken ergodicity by necessity leads to entropy loss and the approach of zero values of entropy for T→0 consequently lack any foundation.

Johari [36] employed “*thermodynamics of lattice vacancies to test the merits of the view that (i) statistical entropy, $k_{B}\ln\Omega$, vanishes on vitrification of a liquid and hence there is no residual entropy and (ii) $k_{B}\ln\Omega$ of a non-ergodic state would increase with time t as its structure relaxes. We argue that this view conflicts with the precepts of the configurational entropy of a crystal, −R[xlnx+(1−x)ln(1−x)], where x is the fractional population of vacancies, and with the observed decrease in x with t on structural relaxation*”. We further elaborate on this argumentation in Section 3.2.

Johari draws in [36] attention to the increased vapor pressure and solubility of glasses as a direct indication of the existence of a residual entropy. This analysis is further advanced in [111]. Aji and Johari et al. [39,112,113,114] also analyzed the effect of irreversible flow processes on the residual entropy determination. The general conclusion is that “*spontaneous enthalpy release has little effect on the entropy change determined from the $C_{p}d\ln\left(T\right)$ integral and, contrary to recent suggestions, $S_{res}$ is real*”, as stated in [39], or that “*these measurements also show that violation of the Clausius theorem is relatively inconsequential for interpreting the entropy of the glassy state*”, as noted in [114] and references therein.

The point of view of the present authors on this topic is described in detail in the monographs [24,68,69] and in a variety of papers on this and related topics ([35,49,82,115,116,117,118] and references cited therein). We do not repeat them here in detail. We would like only to mention that we fully retain the conventional point of view, reflecting directly the thermodynamic nature of glasses as first described by Simon, and that there exists a variety of glass properties (thermodynamic functions and thermodynamic coefficients, solubility, vapor pressure, reactivity, etc.) that cannot be understood appropriately in terms of the entropy-loss assumption. In the subsequent section, we underline these statements, discussing a very instructive model showing that the classical approach to the determination of the residual entropy is the correct approach.

### 3.2. Residual Entropy: A Simple Model

For the analysis of the thermodynamic and kinetic properties of glass-forming melts, in [24], a simple lattice-hole free-volume statistical model was advanced and employed. It is, on one hand, simple enough to allow a variety of conclusions and, on the other hand, it is sufficiently correct to appropriately describe the respective properties. In the framework of this model, the structural-order parameter is introduced as the ratio of the number of holes, $n_{hole}$, divided by the sum of the number of particles of the liquid, $n_{liquid}$, and the number of holes, that is, $\xi =n_{hole}/\left(n_{liquid}+n_{hole}\right)$. This model we utilize here to demonstrate the essence of one of the solutions of the problem of the existence or non-existence of the residual entropy reviewed in the preceding section. A similar model has been employed by Johari [36] in the analysis of configurational and residual entropies of disordered crystals and the entropy’s behavior on the glass formation discussed above.

According to this model, the configurational contributions per mole to the thermodynamic functions (*H*: enthalpy; *S*: entropy; *G*: Gibbs free energy) are given by the following relations [24,116]:

(5) $$H_{conf}\left(\xi \right)≅\chi RT_{m}\xi \;,\;S_{conf}≅-R\left(\ln\left(1-\xi \right)+\frac{\xi }{1-\xi }\ln\xi \right)\;$$

$$G_{conf}≅\chi RT_{m}\xi +RT\left(\ln\left(1-\xi \right)+\frac{\xi }{1-\xi }\ln\xi \right)\;$$

Here *R* is the universal gas constant and $T_{m}$ is the melting temperature of the liquid under consideration. The parameter χ we set in the computations here to be equal to χ=3. The equilibrium value of the structural-order parameter, $\xi_{e}$, is obtained as

(6) $$\frac{{\left(1-\xi_{e}\right)}^{2}}{\ln\xi_{e}}=-\frac{1}{\chi }\left(\frac{T}{T_{m}}\right)\;$$

A substitution of $\xi_{e}$ into the expression for the configurational entropy yields its equilibrium value, $S_{conf}^{\left(e\right)}$, that is, the configurational entropy of the metastable liquid.

**Figure 2:**
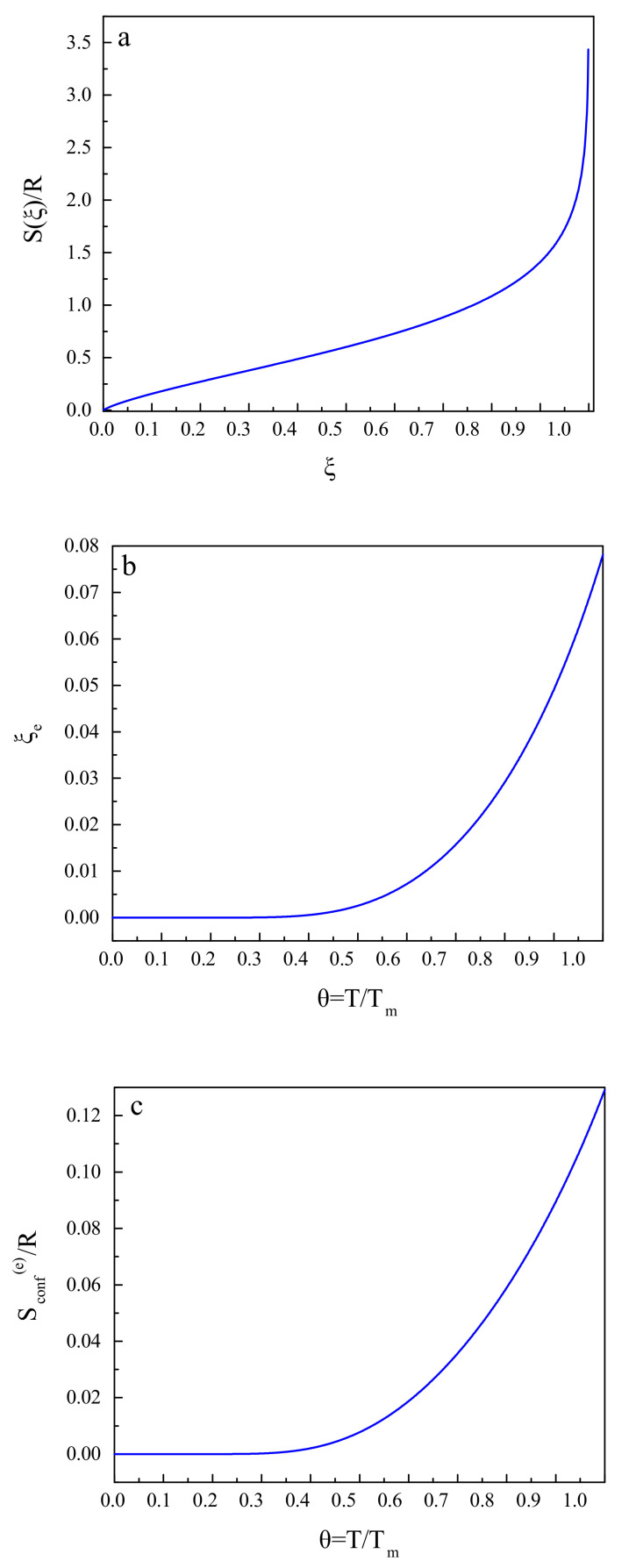
(**a**) Dependence of the configurational entropy, $S_{conf}$, on the structural-order parameter, ξ, according to Equation (5). (**b**) Equilibrium value, $\xi_{e}$, of the structural-order parameter as a function of reduced temperature, $\theta =(T/T_{m})$, according to Equation (6). (**c**) Equilibrium value of the configurational entropy, $S_{conf}^{\left(e\right)}$, as a function of the reduced temperature, $T/T_{m}$, according to Equations (5) and (6).

These relations and results are illustrated in Figure 2. Figure 2a shows the dependence of the configurational entropy, $S_{conf}$, on the structural-order parameter, ξ. Figure 2b gives an illustration of the equilibrium value, $\xi_{e}$, of the structural-order parameter as a function of reduced temperature, $\theta =(T/T_{m})$, and Figure 2c supplies us with the same dependence for the equilibrium value of the configurational entropy, $S_{conf}^{\left(e\right)}$.

The value of the structural-order parameter established in the considered cooling and heating processes depends on the cooling and heating rates. We assume here cyclic cooling and heating proceeding with the same absolute value of the rate of change, $q_{\theta }=d\left(T/T_{m}\right)/dt$, of the reduced temperature, $\left(T/T_{m}\right)$. The relaxation time we suppose to be of the Vogel–Fulcher–Tammann form [24] given by

(7) $$\tau \left(T,p\right)=\frac{h}{k_{B}T}\exp\left(\frac{A}{T-T_{0}}\right)\;$$

Here, *h* is Planck’s constant, and $T_{0}$ and *A* are chosen as $T_{0}=T_{m}/2$ and $A=5RT_{m}$ [116].

**Figure 3:**
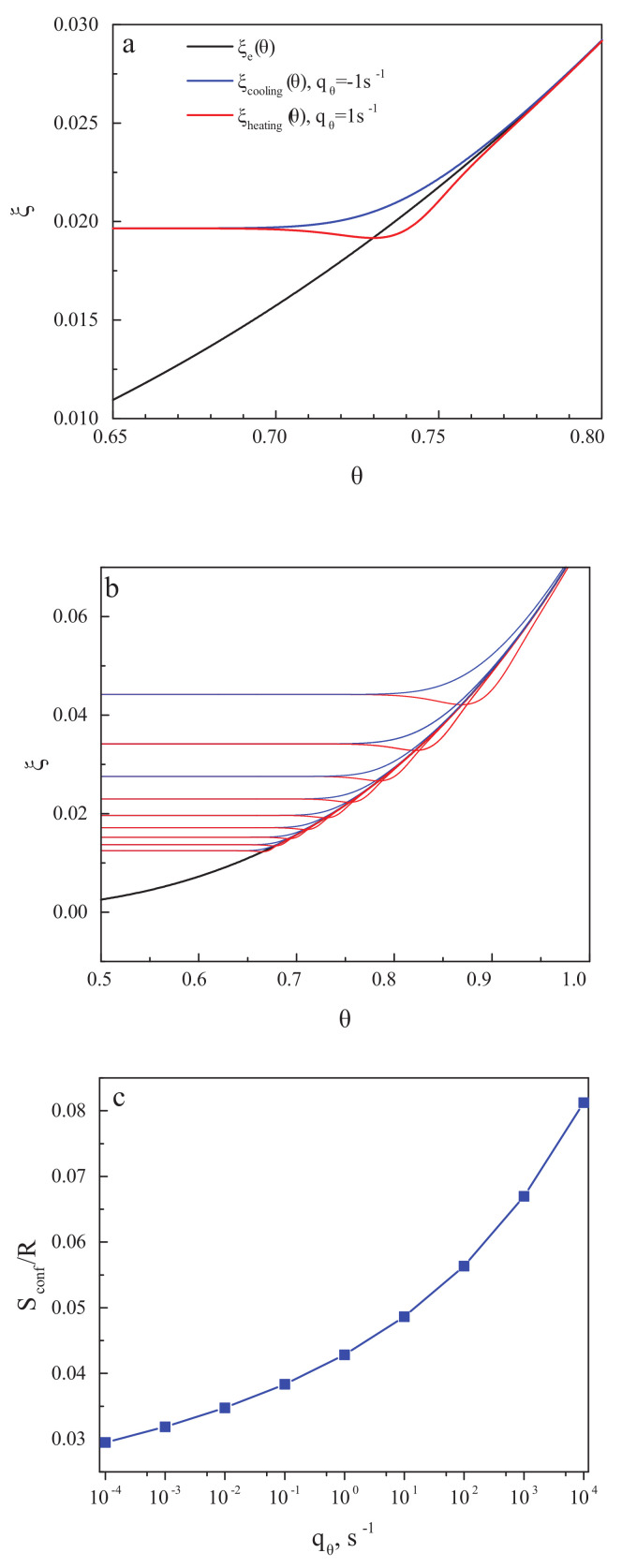
Dependence of the structural-order parameter, ξ, on the reduced temperature, $\theta =(T/T_{m})$, for (**a**) one particular and (**b**) some set of heating and cooling rates. In cooling, the ξ=ξ(θ) curve decreases monotonically with decreasing temperature and approaches a constant value corresponding to the frozen-in state of a glass. The respective values of the residual or frozen-in configurational entropy are shown in dependence on cooling rate in (**c**). The results are in full agreement with the ideas concerning non-zero values of the residual entropy in vitrification as developed in the last century and reconfirmed by the discussion reviewed in the preceding section.

Figure 3a,b shows the structural-order parameter, ξ, as a function of reduced temperature, $\theta =(T/T_{m})$, for different values of the cooling and heating rate. In cooling, the ξ=ξ(θ) curve decreases monotonically with decreasing temperature and approaches a constant value corresponding to the frozen-in state of a glass. The respective value of the residual or frozen-in configurational entropy is shown in the dependence on the cooling rate in Figure 3c. The results are in full agreement with the ideas developed in the last century. The results for the residual entropy do depend exclusively on the temperature course (determining the value of the relaxation time). They are not affected by the existence or absence of irreversible processes.

We check finally whether, as supposed by Reiss [91], irreversible processes may lead to some misinterpretation of data if irreversible processes take place in the measurements of the specific heats being the basic quantity for the determination of the entropy of a given system. Of course, in the glass transition interval, irreversible processes occur that can be measured by the computation of the entropy production. The entropy production can be determined theoretically via the following relation [116]:

(8) $$\frac{d_{i}S}{d\theta }=\frac{1}{Tq_{\theta }\tau }{\left.\left(\frac{\partial^{2}G_{conf}}{\partial \xi^{2}}\right)\right|}_{\xi =\xi_{e}}$$

**Figure 4:**
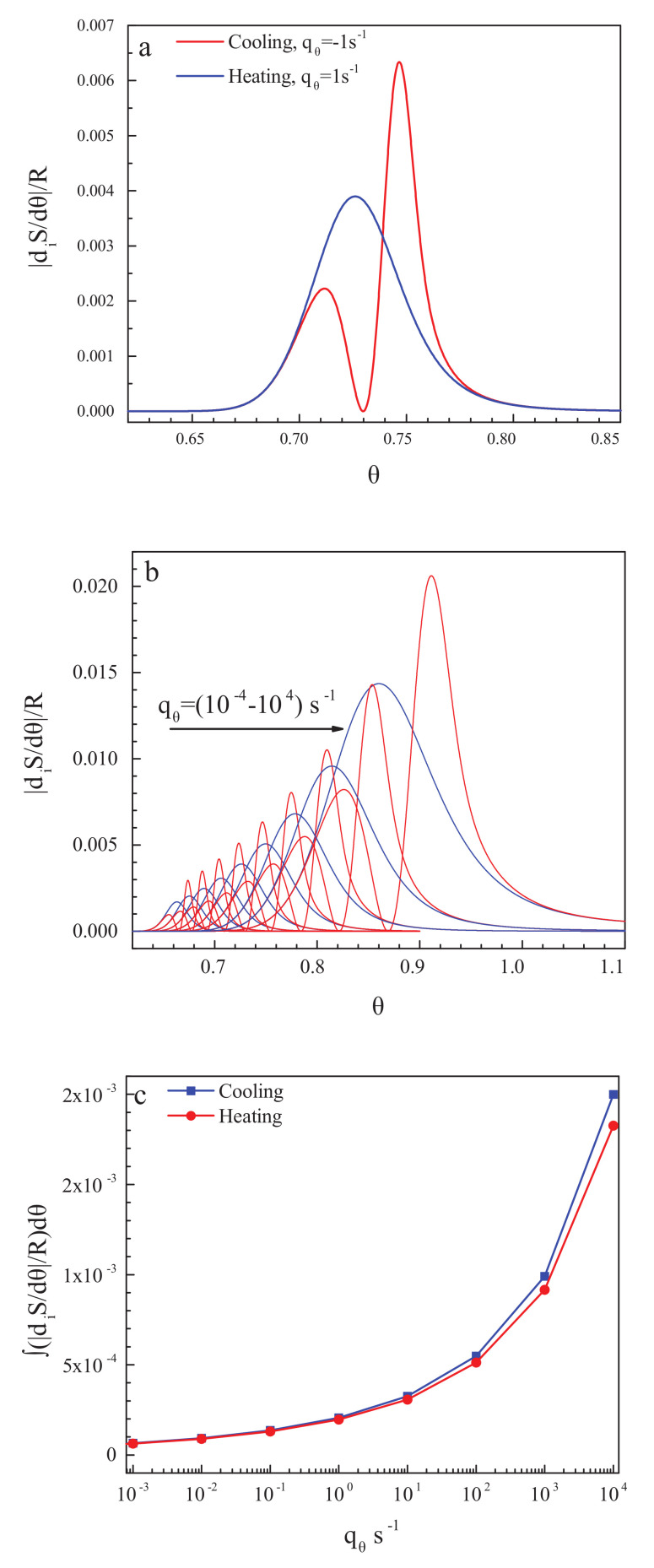
Entropy production, $\left(1/R\right)\left(d_{i}S/d\theta \right)$, in dependence on reduced temperature, $\theta =(T/T_{m})$ for (**a**) one particular and (**b**) some set of cooling and heating rates, $q_{\theta }$. With a decrease in the cooling rate, the glass transition temperature is shifted to lower temperatures [83,84], and the width of the glass transition region decreases [86]. In addition, in (**c**), the total entropy produced in the respective cooling and heating runs is shown in dependence on the rate of change of temperature.

The results of the computation of the entropy production for the cyclic processes discussed above are presented in Figure 4a,b for one particular (Figure 4a) and some set (Figure 4b) of cooling and heating rates. With a decrease in the cooling rate, the glass transition temperature is shifted to lower values of temperature [83,84], and the width of the glass transition region decreases [86]. Below the glass transition region, the entropy production caused by relaxation is equal to zero, and flow processes do not occur there (cf. [52] and its discussion in Section 2.1). Consequently, as expected, neither in the state of a metastable liquid nor in the state of a frozen-in glass, entropy production processes take place. This result underlines once again that glasses do not flow below the glass transition interval of finite width with a perceptible rate at relevant time scales.

In addition, in Figure 4c, the total entropy produced in the respective cooling and heating runs is shown in dependence on the rate of change of temperature. This turns out to be by orders of magnitude smaller than the values of the residual entropy. Already by this reason, it cannot significantly affect the values of the residual entropy determined on the basis of appropriate measurement procedures.

### 3.3. On the Behavior of the Thermodynamic Coefficients in the Glass-Transition Range

The treatment of vitrification as a process of freezing-in the structure of the liquids leads to a variety of consequences, which are in agreement with experimental data. This freezing-in process implies that at the glass transition temperature, the response of a given system changes qualitatively, leading to jumps of the thermodynamic coefficients. This qualitative change of the response is also the origin of the partial similarity (but, to repeat, not the identity [24,74,83,84,87]) of a glass transition (transition of a metastable equilibrium to a frozen-in, non-equilibrium state at a temperature, $T_{g}$, defined by cooling or heating rates) and second-order equilibrium phase transitions with a transition point defined exclusively by thermodynamics (for details, see [24,68,83]). The hysteresis curves for the values of the structural-order parameter lead to specific features of the behavior of thermodynamic coefficients in the transition ranges, supplementing mentioned basic features. Here we would like to address briefly one of these.

In his paper [119], Angell notes that the glass transition temperature may be defined in different ways, resulting in its values differing by several Kelvin or even more (see also Mazurin [120]). In the description of Figure 2 in [119], one can find the intriguing statement that the “$T_{g,onset\;cooling}$ temperature” merges always with the position of the $C_{p}$-overshoot. The “onset cooling” term refers to the intersect between the $C_{p}$ slope of the liquid with the tangent of the heat capacity for cooling at the middle of the liquid–glass transition range with respect to $\Delta C_{p}$. It is also supposed that the similarly defined temperature $T_{g,onsetheating}$ for the heating curve is very close to the mid-point cooling glass transition temperature, $T_{g,mpc}$. These suggestions are supposed to be true only for cyclic measurements, for which equal values of cooling and heating rates are applied. We would like to check here whether these statements can be verified or not by results of calculations within the model of the glass transition as discussed here.

Knowing the dependence of the structural-order parameter, ξ(T), on temperature, the configurational contribution to the heat capacity is given by the following relations [24,82,116]:

(9) $$\frac{C_{p}^{conf}\left(T\right)}{R}=\frac{1}{R}{\left(\frac{\partial H_{conf}}{\partial \xi }\right)}_{p,T}\frac{d\xi }{dT}=\chi T_{m}\frac{d\xi }{dT}$$

We employ here the reduced heat capacity. The reduced heat capacity is obtained from the $C_{p}^{conf}$ dependencies so as to obtain constant values of $C_{p}$ in the liquid and glassy states. Because we know the dependence of the equilibrium values of $\xi_{e}$ on temperature, it can be used for the reduction procedure, employing the relation

(10) $$C_{p,conf}^{\left(red\right)}\left(T\right)=\chi T_{m}\frac{\left(\frac{d\xi }{dT}\right)}{\left(\frac{d\xi_{e}}{dT}\right)}\;$$

**Figure 5:**
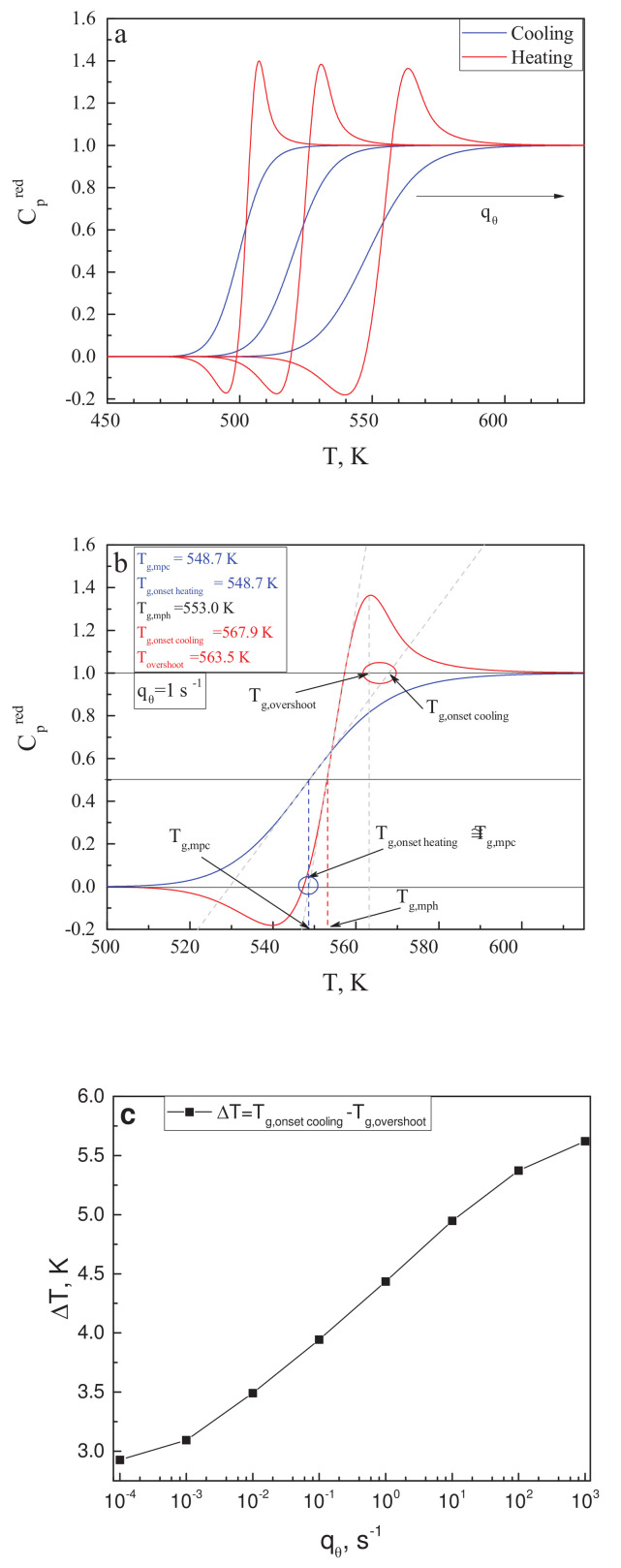
Calculated reduced heat capacity curves for various scanning rates (cooling rate is the same as the heating rate) to clarify the positions of differently defined glass transition temperatures. (**a**) $C_{p}$ curves for three different cooling and heating rates ($q_{\theta }=10^{-4}$, $10^{-2}$, and 1·s${}^{-1}$). (**b**) Visual definition of respective glass transition temperatures obtained: $T_{g,mpc}$, $T_{g,mph}$, $T_{g,overshoot}$, $T_{g,onset\;cooling}$ and $T_{g,onset\;heating}$. (**c**) Calculated difference ΔT between $T_{g,onset\;cooling}$ and $T_{g,overshoot}$ for a wider range of rates of change of temperature. A nearly linear dependence is observed.

Utilizing the data for ξ(T) as shown in Figure 3b, the reduced heat capacity curves have been calculated for different values of $q_{\theta }$ in the range from $10^{-4}$·s${}^{-1}$ up to $10^{3}$·s${}^{-1}$. Some of the results are shown in Figure 5a. Following the procedures as described in [119], in the caption to Figure 2 there, we performed our own estimates of the following quantities: the mid-point heating temperature, $T_{g,mph}$; the mid-point cooling temperature, $T_{g,mpc}$; and the position of the overshoot temperature, $T_{g,overshoot}$, of the onset cooling temperature, $T_{g,onset\;cooling}$, and of the onset heating temperature, $T_{g,onset}$. This procedure is illustrated in Figure 5b for the scanning rate of $q_{\theta }$ = 1·s${}^{-1}$. The blue circle in Figure 5b encloses the area in which the comparison of $T_{g,onset}$ and $T_{g,mpc}$ is performed; the red circle does the same for $T_{g,overshoot}$ and $T_{g,onset\;cooling}$.

From Figure 5b, it is evident that for a single scanning rate, $T_{g,onset}≅T_{g,mpc}$ is satisfied, while the values of $T_{g,overshoot}$ and $T_{g,onset\;cooling}$ vary by several Kelvin. To further explore this result, similar estimates have been made for seven different values of $q_{\theta }$ in the mentioned range of calculations. We found that the difference between $T_{g,onset}$ and $T_{g,mpc}$ was always less than 0.5 K; thus the respective suggestion in [119] is justified within our model approach. On the other hand, the difference $\Delta T=T_{g,onset\;cooling}-T_{g,overshoot\;}$ is always relatively large and grows with increasing scanning rate. This dependence is exemplified in Figure 5c. We come consequently to the conclusion that the latter suggestion by Angell is not confirmed by our model approach and may not hold, in general, for different glass-forming liquids, as compared to those studied in [119].

Of course, the analysis performed by us is concentrated on the configurational contributions to the thermodynamic coefficients. In general, these have to be supplemented by vibrational contributions. However, as we believe, no reasonable change in the vibrational contribution to $C_{p}$ with temperature would make our finding consistent with Angell’s conclusion.

## 4. Is the Kauzmann Paradox Really in Conflict with Basic Laws of Nature?

Another topic of intensive current debate connected with entropy variations in the course of the cooling of glass-forming melts and the glass transition is the behavior of the specific entropy difference, Δs, between liquids and crystals denoted commonly as Kauzmann paradox [24,52,121,122,123]. As mentioned by Cahn [121], “*It is rare indeed for a scientific paper to remain central to current concerns several decades after its publication …*” and an “*…increasing number of physicists …keep coming back to Kauzmann and his eponymous paradox …*”. The Kauzmann paradox and its possible consequences have been reanalyzed in detail in a preceding paper [64]; here we would like to review briefly some of its main results, adding some more details.

**Figure 6:**
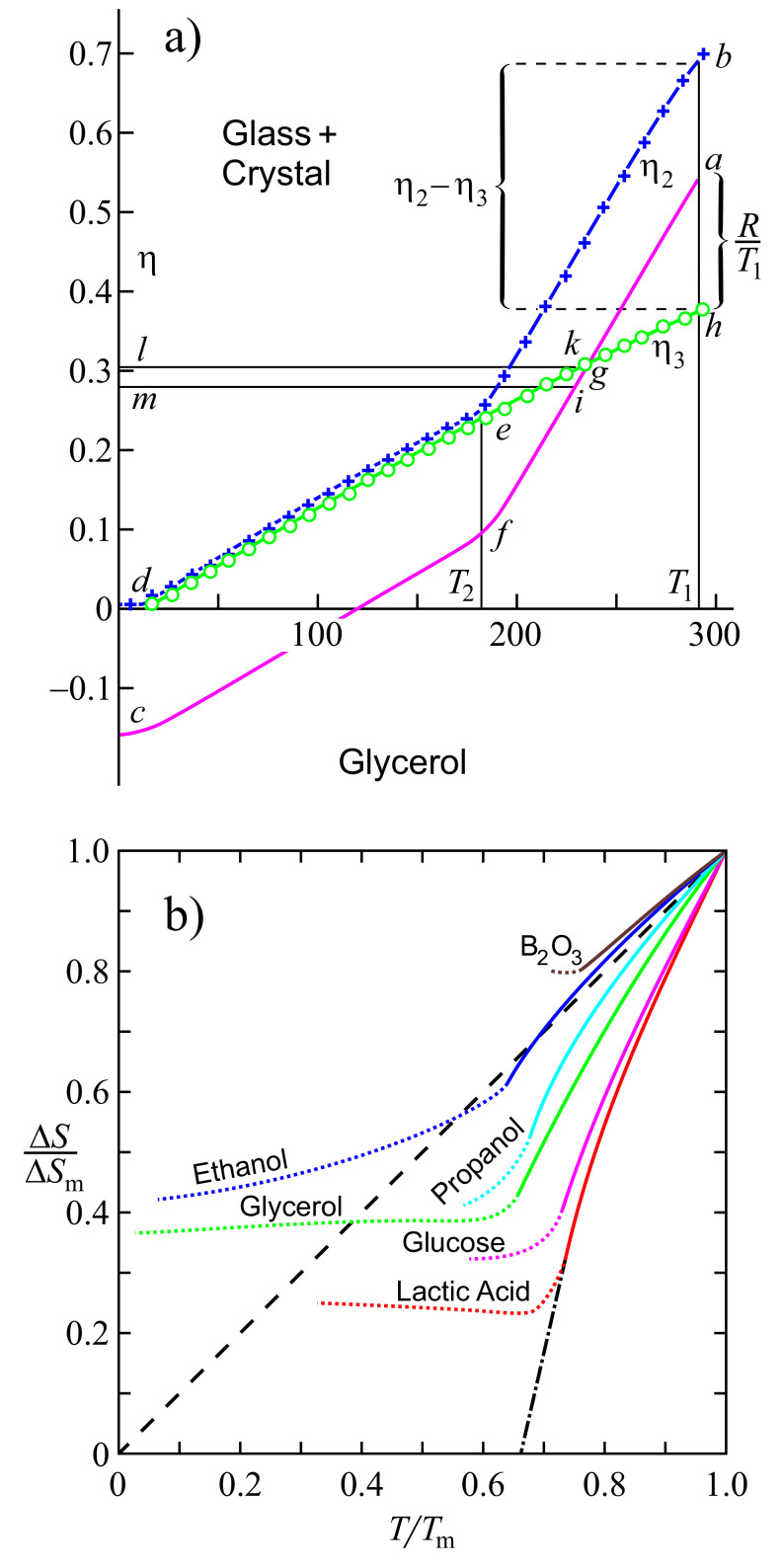
Specific entropy difference between metastable liquids and crystals given in dependence on temperature. (**a**,**b**) is adopted from the papers by Tammann (Figure 4 in [27]) and Kauzmann (Figure 4 in [124]). $\Delta s_{m}$ is the heat of melting or fusion.

As suggested first by Tammann [27,28] and about two decades later by Kauzmann [124], specific entropy differences between liquids and crystals decrease with decreasing temperature and may become equal to zero at a temperature denoted today as the Kauzmann temperature, $T_{K}$, or even less than zero below $T_{K}$ (see Figure 6). Tammann did not consider such a type of behavior as being in conflict with basic laws of physics, supposing that below the Kauzmann temperature, the crystallites should transform into a glass. Additionally, Kauzmann did not see any principal problem in this respect, noting in a footnote in his paper, “*Certainly, it is unthinkable that the entropy of the liquid can ever be very much less than that of the solid. It could conceivably become slightly less at finite temperatures because of a ‘tighter’ binding of the molecule in the highly strained liquid structure…*”. Perhaps this may be the main reason that he was not so happy with the notation “Kauzmann paradox” introduced by Angell (for details, see [125,126]). In line with such considerations, Dyre posed in [44] the question of how seriously the Kauzmann paradox has to be taken and gave some examples in which a crossing of the entropy curves is realized in nature.

Despite his comment in the footnote of his paper [124], Kauzmann in detail discussed possibilities to avoid the crossing of the entropy curves and negative values of Δs below the Kauzmann temperature. This discussion has been continued intensively until now (see [64] for details). As a possible mechanism, he supposed the existence of intensive crystallization near to the Kauzmann temperature connected with the existence of a “pseudo-spinodal curve”. He wrote the following: “*Suppose that when the temperature is lowered a point is eventually reached at which the free energy barrier to crystal nucleation becomes reduced to the same height as the barriers to the simpler motions…At such temperatures the liquid would be expected to crystallize just as rapidly as it changed its typically liquid structure to conform to a temperature or pressure change in its surroundings …There are good theoretical reasons for believing in the existence of such a ‘pseudo-critical temperature’*”. As evident from this statement, Kauzmann supposed the pseudo-spinodal curve to be reached if the Maxwellian relaxation time, $\tau_{R}$, becomes equal to the average time of formation of the first supercritical nucleus, 〈τ〉, that is, if the relation

(11) $$\tau_{R}≅\langle \tau \rangle$$

is satisfied. He supposed this condition, resulting in intensive crystallization, to be fulfilled near to the Kauzmann temperature.

As also can be traced from the quotation given above, Kauzmann connected the rate of crystallization with the average time of formation of the first supercritical nucleus at steady-state conditions with a steady-state nucleation rate, $J_{ss}$. Only in this case is the average time, 〈τ〉, of formation of the first supercritical crystallite correlated with the work of critical cluster formation (the energy barrier to crystal nucleation), $W_{c}$, via [127,128,129]:

(12) $$\langle \tau \rangle ={\langle \tau \rangle }_{ss}≅\frac{1}{J_{ss}V}\;,\;J_{ss}=J_{0}\exp\left(-\frac{W_{c}}{k_{B}T}\right)\;$$

Here *V* is the volume of the melt, $J_{0}$ is a factor determined by the kinetics of crystal nucleation, $k_{B}$ is the Boltzmann constant, and *T* is the absolute temperature.

In order to illustrate the consequences of this preposition, we adopt here the Adam–Gibbs model for viscosity, identifying Δs with the specific entropy difference of liquid and crystal phases. In this case, the temperature of the divergence of viscosity—as frequently supposed to be the case—is identical to the Kauzmann temperature. As outlined in detail in [64], the general consequences are independent of this particular assumption. Accounting for the Maxwell relation [24]:

(13) $$\tau_{R}≅\frac{\eta d_{0}^{3}}{k_{B}T}\;$$

we obtain

(14) $$\tau_{R}≅\tau_{0}\exp\left(\frac{A}{T\Delta s}\right)\;$$

Here $d_{0}$ is a measure of the size of the ambient phase particles in the liquid, η is the viscosity of the liquid, and *A* is a parameter specific for the system under consideration. A combination of Equations (11) and (12) yields the following in the limit Δs→0 or $T\to T_{K}$:

(15) $${\left.J_{ss}V≅\frac{1}{{\langle \tau \rangle }_{ss}}≅\frac{1}{\tau_{R}}≅\frac{1}{\tau_{0}}\exp\left(-\frac{A}{T\Delta s}\right)\right|}_{\Delta s\to 0}\;⟹\;J_{ss}\left(T\to T_{K}\right)=0\;$$

At the Kauzmann temperature, the steady-state nucleation rate tends to zero; intensive crystal nucleation as suggested by Kauzmann, consequently, does not occur at his supposed pseudo-spinodal curve.

In a similar analysis, instead of ${\langle \tau \rangle }_{ss}$, Angell et al. [130] identified the characteristic time of crystallization with the time-lag in nucleation, $\tau_{n}$. As already demonstrated in [64,129], this estimate is, in the range of temperatures Kauzmann was interested in, a much better approximation for the average time of formation of the first supercritical nucleus as compared to ${\langle \tau \rangle }_{ss}$. The time-lag can be determined via Equation (16) [24,64,129]:

(16) $$\tau_{n}≅\omega \frac{\eta d_{0}}{\sigma }n_{c}^{2/3}≅C\tau_{R}n_{c}^{2/3}\;,\;C=\frac{\omega k_{B}T}{\sigma d_{0}^{2}}$$

In Equation (16), ω may vary in the range from 1 to 4 in dependence on the theory employed for the determination of the time-lag, and σ is the specific interfacial energy. With estimates of the parameter *C* as given in [130] in its discussion ($C≅10^{2}-10^{3}$; similar estimates are obtained also in [24]), the mentioned authors arrived at the conclusion that Kauzmann’s condition, Equation (11), cannot be fulfilled, and for this reason, a pseudo-spinodal is absent in melt-crystallization. This result was taken as the starting point for the search of alternative mechanisms to prevent the Kauzmann paradox, such as the concept of an ideal glass transition. However, as is demonstrated below (see also [64]), such additional mechanisms are not required for the resolution of the Kauzmann paradox.

In the estimates of the parameter *C* by Angell et al. [130] and also in [24], the capillarity approximation was employed. More correct estimates involving a size dependence of the specific interfacial energy result, however, in different values of the parameter *C*, allowing the fulfilment of Kauzmann’s condition, Equation (13). In addition, as shown in [129], for isothermal conditions, the average time of formation of the first supercritical nucleus is, in a good approximation, equal to the sum of the induction time, $\tau_{ind}$, widely equal to the time-lag, $\tau_{n}$, and average time of formation of a critical nucleus at steady-state conditions ${\langle \tau \rangle }_{ss}$:

(17) $$\langle \tau \rangle ≅{\langle \tau \rangle }_{ss}+\tau_{ind}$$

**Figure 7:**
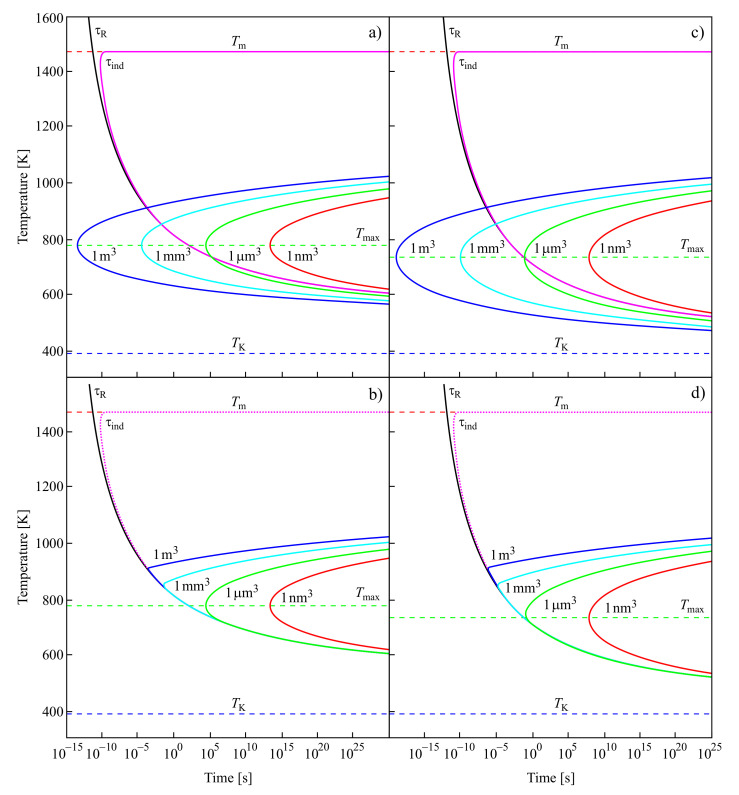
Structural relaxation time, $\tau_{R}$; the induction time, $\tau_{ind}$, required to establish steady-state nucleation; and the average time of formation of a supercritical cluster at steady-state nucleation conditions, ${\langle \tau \rangle }_{ss}≅1/\left(J_{ss}V\right)$, are shown in dependence on temperature (**a**,**c**). The average time of formation of the first supercritical nucleus, 〈τ〉, can be expressed generally in a good approximation as the sum $\langle \tau \rangle ≅{\langle \tau \rangle }_{ss}+\tau_{ind}$ with $\tau_{ind}≅\tau_{n}$ [129]. The dependence of 〈τ〉 on temperature is illustrated in the lower part of (**b**,**d**). For temperatures near to the melting temperature, 〈τ〉 is always determined by ${\langle \tau \rangle }_{ss}$. At the intersection of ${\langle \tau \left(T\right)\rangle }_{ss}$ with $\tau_{ind}\left(T\right)$, 〈τ(T)〉 becomes dominated by the values of $\tau_{ind}$. Values of the parameters employed in the computation of the nucleation rates and related quantities are taken for $2{\mathrm{Na}}_{2}O\cdot 1\mathrm{CaO}\cdot 3{\mathrm{SiO}}_{2}$ [131,132]. In (**b**,**d**), the Vogel temperature is replaced by the Kauzmann temperature (see also text).

For the low-temperature range Kauzmann had in mind, it is nearly equal to the time-lag; for higher temperatures, it is determined by the time of formation of the first supercritical nucleus at steady-state conditions. These results are illustrated in Figure 7.

In the upper curves of Figure 7a,c, the structural relaxation time, $\tau_{R}$; the induction time, $\tau_{ind}$, required to establish steady-state nucleation; and the average time of formation of a supercritical cluster at steady-state nucleation conditions, ${\langle \tau \rangle }_{ss}≅1/\left(J_{ss}V\right)$, are shown in dependence on temperature. The induction time, $\tau_{ind}$, is, except for in the immediate vicinity of the melting temperature, $T_{m}$, practically identical in the relevant time scales to the structural relaxation time. It does not depend on the volume of the system in which crystallization may take place.

In contrast to $\tau_{ind}$, the average time of formation of a supercritical crystal cluster at steady-state nucleation conditions, ${\langle \tau \rangle }_{ss}≅1/\left(J_{ss}V\right)$, does depend on the volume of the system. For small volumes, there may be no intersection of the temperature dependencies of $\tau_{R}\left(T\right)$ and ${\langle \tau \rangle }_{ss}\left(T\right)$, as is evident from the figure. However, there always exists a certain value of the volume of the system at which such crossing occurs. Following the interpretation of Kauzmann, one has then to suppose that the existence and, if it exists, the location of the pseudo-spinodal depend on the volume of the system. Moreover, these would be located not at temperatures near to the Kauzmann temperature but at temperatures higher than the temperature of the maximum of the steady-state nucleation rate. Provided the average time of formation of the first supercritical nucleus is estimated via Equation (12) assuming steady-state nucleation, then there exists always a value of the volume of the system at which this condition is fulfilled. It follows as an additional conclusion that the fulfillment of Equation (11) at steady-state nucleation conditions also does not necessarily result in intensive nucleation. For example, if this condition is fulfilled only in a system with a very large volume, then the fulfillment of Equation (11) does not imply intensive nucleation.

The dependence of 〈τ〉 on temperature is illustrated in the lower part of Figure 7b,d. For temperatures near to the melting temperature, 〈τ〉 is always determined by ${\langle \tau \rangle }_{ss}$. At the intersection of ${\langle \tau \left(T\right)\rangle }_{ss}$ with $\tau_{ind}\left(T\right)$, 〈τ(T)〉 becomes dominated by the values of $\tau_{ind}$. Again, this switch in the quantity dominating the value of 〈τ(T)〉 does not take place at temperatures near to the Kauzmann temperature but at temperatures higher than the temperature of the maximum of the steady-state nucleation rate, $T_{max}$.

For the curves shown in Figure 7a,b, the values of the parameters employed in the computation of the nucleation rates and related quantities are taken for $2{\mathrm{Na}}_{2}O\cdot 1\mathrm{CaO}\cdot 3{\mathrm{SiO}}_{2}$ [131,132]. These parameters are briefly summarized also in [64], where they are employed for the description of similar dependencies in a different form (Figure 6 there). In addition, here in the computations leading to Figure 7c,d, the Vogel temperature is replaced in the equation for the description of the viscosity by the Kauzmann temperature, $T_{K}$. As mentioned earlier, the Kauzmann temperature can be determined from the condition of the maximum of the thermodynamic driving force [65]. As is evident, this replacement leads only to minor changes in the behavior.

The above discussion is performed under the assumption that the liquid is brought very fast to the respective temperature, *T*. However, in reality, processes of clustering may take place already in the course of the cooling process. The account of such prehistory effects may further reduce the time of formation of the first supercritical nucleus. This reduction is particularly significant in the range of temperatures near to the glass transition temperature, respectively, near to the maximum of the steady-state nucleation rate (Figure 7 in [64]). Consequently, the crossing of the 〈τ(T)〉 and $\tau_{R}\left(T\right)$ curves, if taking places, is favored to occur in this range of temperatures and not near to the Kauzmann temperature. However, in any of these cases, the average time of formation of the first supercritical nucleus is either proportional to or widely determined by the relaxation time. This relaxation time diverges in the approach of the Kauzmann temperature, preventing any crystal nucleation (for details, see [64]). Consequently, in the range of temperatures in which Kauzmann expected the pseudo-spinodal to be located, independently of whether or not the condition of Equation (11) is fulfilled, intensive crystal nucleation does not take place.

This conclusion refers not only to Kauzmann’s original suggestion concerning the location of the pseudo-spinodal curve. Additionally, beyond the near vicinity of the Kauzmann temperature, his assumption does not hold. It is not the pseudo-spinodal defined via Equation (11) that governs the maximum of crystal nucleation. In agreement with experimental investigations performed first by Tammann [63] and confirmed by all subsequent experimental and theoretical analysis of crystal nucleation [24,61], there exists one and only one maximum of the steady-state nucleation rate located near to the traditionally defined glass transition temperature (identifying the glass transition temperature with values of the viscosity in the order of $\eta ≅10^{12}$ Pa·s [40]). It is determined by the interplay of the decrease in the work of critical cluster formation and the increase in viscosity with decreasing temperature [60,64,133,134,135]. The location and magnitude of this maximum nucleation rate or the maximum of the overall crystallization rate are not determined by Equation (11), but by other relations both for crystallization caused by variations of temperature or pressure (see [60,133]).

A schematic representation (in discussing Kauzmann’s suggestion of the existence of a pseudo-spinodal curve) of the average structural relaxation time of a supercooled liquid and the average nucleation time is given in Figure 2 in [52], assuming steady-state nucleation to hold. Is is stated that another “*vital concept related to supercooled liquids, which is not well-known within the glass research community, is the liquid stability limit or kinetic spinodal temperature…*” Here, “*the supercooled liquid becomes unstable against crystallization and crystal growth immediately proceeds*”. The shape of the curves for the average time of formation of the first supercritical nucleus presented in their Figure 2 is quite different from our computational results shown in Figure 7, and the dependence of ${\langle \tau \left(T\right)\rangle }_{ss}$ on the volume of the system is also not accounted for. It is correctly noted in the further discussion that sufficiently above $T_{g}$, steady-state nucleation dominates. Consequently, employing this condition of steady-state nucleation in their Figure 2, they implicitly expect a location of the pseudo-spinodal at temperatures above $T_{g}$. In such an interpretation, Kauzmann’s suggestion underlies a direct experimental proof. Existing experimental data do not exhibit any peculiarities indicating the existence of a pseudo-spinodal curve above $T_{g}$. We note also that under such conditions, its location should depend on the volume of the system.

In the further discussion of these topics in [52], our result $\langle \tau \rangle ≅{\langle \tau \rangle }_{ss}+\tau_{ind}$ with $\tau_{ind}≅\tau_{n}$, derived in [129], is adopted partly for the analysis of Kauzmann’s suggestions at low temperatures. However, as already noted, even if critical nuclei would be formed intensively, they would not grow, as the maximum of the growth rate is located at much higher temperatures compared to the maximum of the nucleation rates [60]. This is the origin for why Tammann’s two-stage development method is so widely employed in the analysis of crystallization processes [24,61,136]. However, they also do not form with the intensity supposed by Kauzmann at the low temperatures al supposed by him because of the very low values of the kinetic coefficients, which may also become equal to zero, as discussed in connection with flow processes in glasses.

Summarizing our point of view, we come to the following conclusions: Independent of the results of the estimates of 〈τ〉 and whether this condition can be fulfilled or not, Kauzmann’s suggestion concerning the possibility of the existence of a pseudo-spinodal causing intensive crystal nucleation does not hold by the following reasons derived above: (i) The values of the kinetic parameters are too low and prevent any crystallization near to the Kauzmann temperature. (ii) The maximum nucleation, growth, and overall crystallization rates are defined by other relations. Consequently, the pseudo-spinodal and the properties assigned to it by Kauzmann are not a “*vital concept related to supercooled liquids*” but are irrelevant with respect to the crystallization behavior of glass-forming melts, at least, in the sense that Kauzmann assigned to them.

By the above argumentation, it also follows that Kauzmann’s suggestion concerning the existence of a pseudo-spinodal in melt-crystallization is not sufficient–as he believed—to avoid the realization of the Kauzmann paradox. However, there exists another mechanism preventing this and, much more importantly, preventing also possible contradictions to the third law of thermodynamics. Indeed, according to the general kinetic criterion of glass formation, Equation (3), the rate of cooling determines the value of the glass transition temperature, $T_{g}$, where the metastable liquid is frozen-in into a glass. Assuming a behavior of the relaxation time as given by Equation (14), for any finite value of the rate of change of temperature, the glass-forming melt transforms to a glass at temperatures $T_{g}>T_{K}$. The Kauzmann temperature can be reached only in the limit of zero cooling rates. Such a process cannot be realized in an experiment. The same conclusions can be drawn also by employing other physically reasonable assumptions concerning the temperature dependence of the viscosity and the relaxation time, as discussed in detail in [64].

These conclusions are in full agreement with statements by Simon, who noted already in 1931 that, in cooling, the glass-forming liquids either crystallize or save themselves from crystallization by going over into the vitreous state [32]. This is the mechanism for the prevention of contradictions with the third law of thermodynamics, the only consequence of Tammann’s and Kauzmann’s observations on the behavior of specific entropy differences, which could lead to conflicts with basic laws of nature. Consequently, Simon in fact resolved the Kauzmann paradox about 20 years prior to its formulation. We note also that Simon considered the transition of the melt into a glass as a final process not supplemented by any subsequent crystallization. Consequently, also according to Simon, crystallization is not the ultimate fate of a glass (cf. [52]).

## 5. Summary of Results and Discussion

The present paper is devoted to different aspects concerning the behavior of entropy and quantities depending on it in the transformation of a liquid into a glass and vice versa. In order to proceed in this direction, in Section 2, the basic features characterizing a glass and the glass transition are summarized, and some newly advanced proposals concerning the definition of a glass are critically discussed. As we believe, neither the features (i) that glasses should continuously relax towards the liquid state, or (ii) that their ultimate fate is to be transformed to a crystal we consider as reasonable extensions of the standard definition of glasses and the glass transition advanced by Simon. In addition, a review is given in Section 2 on the advantages and limitations of the different kinetic criteria of glass formation employed here in the analysis of the residual entropy of glasses and the Kauzmann paradox. It is shown that a general criterion of vitrification can be formulated, containing as special approximate cases, all previously formulated particular proposals.

As shown in Section 3, in the review of investigations concerning the concept of residual entropy performed by a variety of authors, the final state of glass in cooling does not depend or only weakly depends on irreversible processes, and entropy production terms are small in comparison with the configurational or frozen-in values of entropy, leading, consequently, only to minor effects in its determination. By these and all other reasons briefly summarized above, we do not consider it as possible to find some compromise reconciling the traditional and the newly advanced entropy-loss concepts as proposed in [137]. Instead, we fully support the conclusion derived by Johari [106], already cited above, but worth being repeated as a general resume: “*The premise that glass formation is a process of continuously breaking ergodicity with entropy loss does not merit serious consideration*”.

In order to avoid any misunderstanding, we would like to cite and comment the following statement by Davies and Jones addressing general problems in advancing appropriate theories of the vitreous state and the glass transition:: “*The central problem is to explain in molecular terms the way in which the glass differs from the liquid and the nature of the change from the glass to the equilibrium liquid. In view of the complexity of glass-forming substances we cannot hope for a detailed microscopic theory. However, an important step towards understanding the behavior of glasses was made by Simon in 1930 when he used ideas drawn from statistical mechanics to give a qualitative explanation of the existence of residual entropy at absolute zero ([57], p. 374)*”. This is completely in line with the analysis discussed briefly above of Nemilov on broken-ergodicity concepts in glass science and the mentioned comments of Howard Reiss on statistical models. Hopefully, over the course of time, an approach can be developed that is in line with results of experimental data, approaches such as that employed by Simon, and combinations of thermodynamic and statistical–mechanical modeling as discussed here in Section 3.2.

In Section 4, the Kauzmann paradox is revisited. It is shown that neither in its primary formulation nor in its possible consequences concerning the validity of the third law of thermodynamics can it lead to any conflict with basic laws of nature. Further, it is shown that a pseudo-spinodal as supposed by Kauzmann yielding intensive crystallization does not exist in melt-crystallization. It is also not required in order to prevent any conflicts with basic laws of nature; these are excluded, as noted already by Simon, by conventional crystallization at temperatures, as a rule, significantly above the Kauzmann temperature, or by a conventional glass transition. For this reason, there is no need for the invention of additional mechanisms of prevention of conflicts of the phenomenon first described by Tammann and then by Kauzmann with basic laws of nature.

We note also that Kauzmann distinguished such pseudo-critical states from critical points or states along the spinodal curve. In particular, he wrote the following ([124], p. 248): “*In the past there has been a considerable amount of speculation concerning the existence of a critical point between crystalline and liquid states analogous to the critical point between liquids and gases. No experimental evidence for or against such a critical point has ever been found [138], though there is reason to believe that none is possible (Bernal [139]; but see Frenkel …[140]). It is apparent, however, that the behavior with which we are here concerned has a certain similarity to the behavior at a critical point in that here, as at a true critical point, the free energy barrier between the crystal and the liquid disappears. On the other hand, there is a fundamental difference in that the two states do not really merge and their free energies are decidedly different …so that one cannot go reversibly from the one state to the other without a normal phase change*”. Meanwhile, the absence of a spinodal in one-component melt-crystallization was established by Skripov and Baidakov on the basis of a thorough analysis of experimental data, first in [141] and reconfirmed in [142]. In agreement with these results and theoretical considerations mentioned by Kauzmann and expressed by Bernal [139], we showed in [64] that a spinodal is absent in melt-crystallization also in multicomponent systems, provided the basic assumption of classical nucleation theory, that critical clusters have compositions similar to those of the newly evolving macroscopic crystal phase, is fulfilled. In such cases, the free energy differences between liquids and crystals have finite positive values in the whole range in which crystallization may proceed. The existence of a spinodal curve requires a simultaneous approach of both the thermodynamic driving force and specific surface energy to zero, as both phases become indistinguishable here. Such a condition cannot be realized at the mentioned conditions.

As noted here earlier, the concept of an ideal glass transition has been formulated as an alternative mechanism to prevent the Kauzmann paradox. In such a procedure, different opinions have been advanced concerning the problem of how such an ideal glassy state has to look like [50,122,130,143,144,145]. In [130], this concept of an ideal glass transition and the motivation of its introduction are described in the following way: “*The alternative to Kauzmann’s suggestion is that the liquid, on slow cooling, continues to lose entropy until, as it approaches the crystal value, an equilibrium transition of a higher order type occurs to yield a disordered phase of vanishingly small configurational entropy. Like a crystal polymorph this equilibrium phase could in principle conform to the third law of thermodynamics*”.

From time to time, experimental data on the properties of glass-forming liquids showing some similarity to second-order equilibrium phase transitions are interpreted in favor of the existence of an ideal glass transition. However, as discussed here briefly in Section 3.3 and in detail in the references cited there, the glass transition exhibits a certain similarity to second-order equilibrium phase transitions, showing also a jump of the thermodynamic coefficients in the glass transition range. Consequently, the mentioned data can be similarly treated in favor of a normal glass transition.

We note also that even if Equation (11) does not imply the specification of temperatures with intensive crystal nucleation, it nevertheless affects the crystallization process and the methods of its description. Indeed, frequently it is found experimentally that first glasses relax to the respective metastable state of the melt and then crystallize [43,134,146]. An explanation of this kind of behavior can be attempted to be achieved via the analysis of the question of whether Equation (11) can be realized or not. Beyond this relation derived in terms of classical nucleation theory, an alternative explanation of the mentioned experimental data has been advanced, assuming that the kinetics of the relaxation of the liquid to the metastable state inhibits the formation of supercritical clusters [43,134,146].

However, provided this is not the case and crystal nucleation proceeds prior to relaxation, then, in principle, additional terms have to be accounted for in the specification of the work of critical cluster formation in the determination of the nucleation rate. Indeed, as anticipated earlier in [69,147], deviations of the state of the liquid from metastable equilibrium affect both the thermodynamic driving force and the specific interfacial energy and, consequently, the work of critical cluster formation in crystal nucleation. In such cases, differences of the configurational parts of the Gibbs free energy and entropy between the current non-equilibrium and the metastable equilibrium states of the liquid have to be appropriately incorporated into the specification of the specific surface energy and the thermodynamic driving force of crystallization. This is one of the realizations for the interplay of the glass transition and crystallization to be explored in detail in future studies.

## References

[1] Gibbs J.W. (1906). Graphical Methods in the Thermodynamics of Fluids.

[2] Gibbs J.W. (1905). Elementare Grundlagen der Statistischen Mechanik (Elementary Foundation of Statistical Mechanics).

[3] Landau L.D., Lifshitz E.M. (1980). Statistical Physics.

[4] Koverda V.P., Skokov V.N. (2012). Maximum entropy in a nonlinear system with a (1/*f*)-power spectrum. Physica A.

[5] Feistel R., Ebeling W. (2011). Physics of Self-Organization and Evolution.

[6] Kondepudi D., Prigogine I. (2014). The Second Law of Thermodynamics and the Arrow of Time. Modern Thermodynamics: From Heat Engines to Dissipative Structures.

[7] Feistel R. (2017). Self-organisation of symbolic information. Eur. Phys. J. Spec. Top..

[8] Feistel R., Ebeling W. (2016). Entropy and the Self-Organization of Information and Value. Entropy.

[9] Gujrati P.D. (2015). On Equivalence of Nonequilibrium Thermodynamic and Statistical Entropies. Entropy.

[10] Ebeling W., Kremp D., Parthey H., Ulbricht H. (1970). Reversibilität und Irreversibilität als physikalisches Problem in philosophischer Sicht (Reversibility and irreversibility as a physical problem from a philosophical point of view). Wiss. Z. Univ. Rostock.

[11] Schmelzer J.W.P. (1981). Über eine Möglichkeit der Lösung des Widerspruchs zwischen mikroskopischer Reversibilität und makroskopischer Irreversibilität der Bewegung (On one possibility of resolution of the contradiction between microscopic reversibility and macroscopic irreversibility of motion). Rostocker Philos. Manuskr..

[12] Boltachev G.S., Schmelzer J.W.P. (2010). On the definition of temperature and its fluctuations in small systems. J. Chem. Phys..

[13] Chua Y.Z., Schulz G., Shoifet E., Huth H., Zorn R., Schmelzer J.W.P., Schick C. (2014). Glass transition cooperativity from broad band heat capacity spectroscopy. Colloid Polym. Sci..

[14] Schmelzer J.W.P., Boltachev G.S., Baidakov V.G. (2006). Classical and Generalized Gibbs’ Approaches and the Work of Critical Cluster Formation in Nucleation Theory. J. Chem. Phys..

[15] Nernst W. (1918). Die Theoretischen und Experimentellen Grundlagen des Neuen Wärmesatzes (Theoretical and Experimental Foundation of the New Heat Theorem).

[16] Haase R. (1956). 50 Jahre Nernstscher Wärmesatz (50 years of Nernst’s heat theorem). Die Naturwiss..

[17] Gutzow I.S., Schmelzer J.W.P., Schmelzer J.W.P., Gutzow I.S. (2011). Glasses and the Third Law of Thermodynamics. Glasses and the Glass Transition.

[18] Einstein A. (1914). Beiträge zur Quantentheorie (Contributions to quantum theory). Verh. Deutsch. Phys. Ges..

[19] Simon F. (1937). On the Third Law of Thermodynamics. Physica.

[20] Planck M. (1930). Theorie der Wärme (Theory of Heat).

[21] Planck M. (1954). Vorlesungen über Thermodynamik (Lectures on Thermodynamics).

[22] Bazarow I.P. (1974). Thermodynamik (Thermodynamics).

[23] Nernst W. (1926). Theoretische Chemie (Theoretical Chemistry).

[24] Gutzow I.S., Schmelzer J.W.P. (2013). The Vitreous State: Thermodynamics, Structure, Rheology, and Crystallization.

[25] Nemilov S.V. (1995). Thermodynamic and Kinetic Aspects of the Vitreous State.

[26] Simon F., Lange F. (1926). Zur Frage der Entropie amorpher Substanzen (On the question concerning the entropy of amorphous substances). Z. Phys..

[27] Tammann G. (1930). Die Entropien eines Kristalls und seiner Schmelze in Abhängigkeit von der Temperatur (The entropies of a crystal and its melt in dependence on temperature). Ann. Phys..

[28] Tammann G., Jenckel E. (1930). Die Kristallisationsgeschwindigkeit und die Kernzahl des Glycerins in Abhängigkeit von der Temperatur (Rate of crystallization and the number of nuclei in dependence on temperature). Z. Anorg. Allg. Chem..

[29] Gibson G.E., Giauque W.F. (1923). The Third Law of Thermodynamics. Evidence from the Specific Heats of Glycerol that the Entropy of a Glass Exceeds that of a Crystal at the Absolute Temperature. J. Am. Chem. Soc..

[30] Simon F. (1927). Zum Prinzip on der Unerreichbarkeit des absoluten Nullpunktes (On the principle of unattainability of the absolute zero). Z. Phys..

[31] Simon F. (1930). Fünfundzwanzig Jahre Nernstscher Wärmesatz (Twenty-five years of Nernst’s heat theorem). Ergebnisse der exakten Naturwiss..

[32] Simon F. (1931). Über den Zustand der unterkühlten Flüssigkeiten und Gläser (On the state of undercooled liquids and glasses). Z. Anorg. Allg. Chem..

[33] Wolynes P.G. (1997). Entropy Crises in Glasses and Random Heteropolymers. J. Res. Natl. Inst. Stand. Technol..

[34] Gutzow I., Petroff B., Möller J., Schmelzer J.W.P. (2007). Glass transition and the Third Principle of thermodynamics: Reconsideration of a classical problem. Phys. Chem. Glasses.

[35] Gutzow I., Schmelzer J.W.P. (2009). The Third Principle of thermodynamics and the zero-point entropy of glasses: History and new developments. J. Non-Cryst. Solids.

[36] Johari G.P. (2010). Configurational and residual entropies of nonergodic crystals and the entropy’s behavior on glass formation. J. Chem. Phys..

[37] Gutzow I.S., Petroff B.P., Todorova S.V., Schmelzer J.W.P., Schmelzer J.W.P., Gutzow I.S. (2011). Thermodynamics of Amorphous Solids, Glasses, and Disordered Crystals. Glasses and the Glass Transition.

[38] Pauling L. (1935). The Structure and Entropy of Ice and of Other Crystals with Some Randomness of Atomic Arrangement. J. Am. Chem. Soc..

[39] Aji D.P.B., Johari G.P. (2010). Fictive temperature, structural relaxation, and reality of residual entropy. J. Phys. Chem. B.

[40] Tammann G. (1933). Der Glaszustand (The Vitreous State).

[41] Tammann G., Kohlhaas A. (1929). Die Begrenzung des Erweichungsintervalles der Gläser und die abnorme Änderung der spezifischen Wärme und des Volumens im Erweichungsgebiet (The boundaries of the softening interval of glasses and the abnormal variation of the specific heat and the volume in the softening interval). Z. Anorg. Allg. Chem..

[42] Parks G.S., Huffmann H.M. (1927). Studies on glass. I. The transition between the glassy and liquid states in the case of some simple organic compounds. J. Phys. Chem..

[43] Schick C., Zhuravlev E., Androsch R., Wurm A., Schmelzer J.W.P., Schmelzer J.W.P. (2014). Influence of Thermal Prehistory on Crystal Nucleation and Growth in Polymers. Glass: Selected Properties and Crystallization.

[44] Dyre J. (2006). Colloquium: The glass transition and elastic models of glass-forming liquids. Rev. Mod. Phys..

[45] Wool R.P. (2008). Twinkling fractal theory of the glass transition. J. Polym. Sci. Polym. Phys..

[46] Ojovan M.I. (2013). Ordering and structural changes at the glass-liquid transition. J. Non-Cryst. Solids.

[47] Sanditov D.S. (2012). Model of delocalized atoms in the physics of the vitreous state. J. Exp. Theor. Phys..

[48] Wang Z., Sun B.A., Bai H.Y., Wang W.H. (2014). Evolution of hidden localized flow during glass-to-liquid transition in metallic glass. Nat. Commun..

[49] Tropin T.V., Schmelzer J.W.P., Aksenov V.L. (2016). Modern aspects of the kinetic theory of glass transition. Phys. Usp..

[50] Debenedetti P.G. (1996). Metastable Liquids: Concepts and Principles.

[51] Anderson P.W. (1995). Viewpoint: The future. Science.

[52] Zanotto E.D., Mauro J.C. (2017). The glassy state of matter: Its definition and ultimate fate. J. Non-Cryst. Solids.

[53] Tool A.Q. (1946). Relation between inelastic deformability and thermal expansion of glass in its annealing range. J. Am. Ceram. Soc..

[54] Gutzow I., Grigorova T., Avramov I., Schmelzer J.W.P. (2002). Generic phenomenology of vitrification and relaxation and the Kohlrausch and Maxwell equations. Phys. Chem. Glasses.

[55] Zanotto E.D. (1998). Do cathedral glasses flow?. Am. J. Phys..

[56] Zanotto E.D., Gupta P.K. (1999). Do cathedral glasses flow?—Additional remarks. Am. J. Phys..

[57] Davies R.O., Jones G.O. (1953). Thermodynamic and kinetic properties of glasses. Adv. Phys..

[58] Ojovan M.I. (2012). Viscous flow and the viscosity of melts and glasses. Phys. Chem. Glasses.

[59] Priven A.I., Schmelzer J.W.P., Gutzow I.S. (2011). Methods of Prediction of Glass Properties from Chemical Compositions. Glasses and the Glass Transition.

[60] Schmelzer J.W.P., Abyzov A.S., Fokin V.M., Schick C., Zanotto E.D. (2015). Crystallization in glass-forming liquids: Maxima of nucleation, growth, and overall crystallization rates. J. Non-Cryst. Solids.

[61] Fokin V.M., Yuritsyn N.S., Zanotto E.D., Schmelzer J.W.P. (2006). Homogeneous crystal nucleation in silicate glasses: A 40 years perspective. J. Non-Cryst. Solids.

[62] Schmelzer J.W.P., Abyzov A.S., Fokin V.M., Schick C., Zanotto E.D. (2015). Crystallization in glass-forming liquids: Effects of decoupling of diffusion and viscosity on crystal growth. J. Non-Cryst. Solids.

[63] Tammann G. (1898). Über die Abhängigkeit der Zahl der Kerne, welche sich in verschiedenen unterkühlten Flüssigkeiten bilden, von der Temperatur (On the dependence of the number of nuclei, which are formed in undercooled liquids, on temperature). Z. Phys. Chem..

[64] Schmelzer J.W.P., Abyzov A.S., Fokin V.M., Schick C. (2017). Kauzmann paradox and the crystallization of glass-forming melts. J. Non-Cryst. Solids.

[65] Schmelzer J.W.P., Abyzov A.S., Fokin V.M. (2016). Crystallization of glass: What we know, what we need to know. Int. J. Appl. Glass Sci..

[66] https://en.wikipedia.org/wiki/Amber.

[67] http://geology.com/rocks/obsidian.shtml.

[68] Schmelzer J.W.P., Gutzow I.S. (2011). Glasses and the Glass Transition.

[69] Schmelzer J.W.P. (2014). Glass: Selected Properties and Crystallization.

[70] Berthier L., Ediger M.D. (2016). Facets of glass physics. Phys. Today.

[71] Gibbs J.W. (1928). The Collected Works.

[72] Bartenev G.M. (1949). On the vitrification of high-polymer bodies at periodic deformation. Dokl. Akad. Nauk SSSR.

[73] Bartenev G.M. (1951). On the relation between the glass transition temperature of silicate glass and rate of cooling or heating. Dokl. Akad. Nauk SSSR.

[74] Bartenev G.M., Lukyanov I.A. (1955). Dependence of the glass-transition temperature of amorphous substances on the heating rate and the relation between glass transition temperature and activation energy. Zhurnal Fizicheskoi Khimii.

[75] Ritland H.N. (1954). Density phenomena in the transformation range of a borosilicate crown glass. J. Am. Ceram. Soc..

[76] Volkenstein M.V., Ptizyn O.B. (1955). Relaxation theory of glass transition. Dokl. Akad. Nauk USSR.

[77] Volkenstein M.V., Ptizyn O.B. (1956). Relaxation theory of glass transition: Solution of the basic equation and its analysis. Zhurnal Tekhnicheskoi Fiziki.

[78] Cooper A.R., Gupta P.K. (1982). A dimensionless parameter to characterize the glass transition. Phys. Chem. Glasses.

[79] Bragg W.L., Williams E.J. (1934). The effect of thermal agitation on atomic arrangement in alloys. Proc. R. Soc. Lond. A.

[80] Reiner M. (1964). The Deborah number. Phys. Today.

[81] Mazurin O.V., Cooper A.R. (1985). An analysis of the suitability of the Lillie number to characterize the glass transition of real glass-forming substances. J. Non-Cryst. Solids.

[82] Möller J., Gutzow I., Schmelzer J.W.P. (2006). Freezing-in and production of entropy in vitrification. J. Chem. Phys..

[83] Schmelzer J.W.P. (2012). Kinetic criteria of glass formation and the pressure dependence of the glass transition temperature. J. Chem. Phys..

[84] Schmelzer J.W.P., Tropin T.V. (2015). Kinetic criteria of glass-formation, pressure dependence of the glass-transition temperature, and the Prigogine-Defay ratio. J. Non-Cryst. Solids.

[85] Johari G.P. (2017). On relative merits of the criteria of glass formation and effects of ultraviscous liquid properties. J. Non-Cryst. Solids.

[86] Schmelzer J.W.P., Tropin T.V. (2013). Dependence of the width of the glass transition interval on cooling and heating rates. J. Chem. Phys..

[87] Tropin T.V., Schmelzer J.W.P., Gutzow I., Schick C. (2012). On the theoretical determination of the Prigogine-Defay ratio in glass transition. J. Chem. Phys..

[88] Gujrati P.D. (2010). Nonequilibrium thermodynamics: Structural relaxation, fictive temperature, and Tool-Narayanaswamy phenomenology in glasses. Phys. Rev. E.

[89] Johari G.P. (1997). Determining Temperature-Invariant Enthalpy Change and Other Thermodynamic Functions on Transformation of Proteins and Other Biopolymers. J. Phys. Chem. B.

[90] Kivelson D., Reiss H. (1999). Metastable Systems in Thermodynamics: Consequences, Role of Constraints. J. Phys. Chem. B.

[91] Reiss H. (2009). Apparent entropy, residual entropy, causality, metastability, constraints, and the glass transition. J. Non-Cryst. Solids.

[92] Yang C.N., Lee T.D. (1952). Statistical Theory of Equations of State and Phase Transitions. I. Theory of Condensation. Phys. Rev..

[93] Baidakov V.G., Schmelzer J.W.P., Röpke G., Priezzhev V.B. (2008). On the Thermodynamic Properties of Metastable Systems. Nucleation Theory and Applications.

[94] Baidakov V.G. (1994). Thermophysical properties of superheated liquids. Soviet Technology Reviews.

[95] Davies R.O., Jones G.O. (1953). The irreversible approach to equilibrium in glasses. Proc. R. Soc. A.

[96] Gupta P.K., Mauro J.C. (2009). The configurational entropy of glass. J. Non-Cryst. Solids.

[97] Mauro J.C., Gupta P.K., Loucks R.J., Varshneya A.K. (2009). Non-equilibrium entropy of glasses formed by continuous cooling. J. Non-Cryst. Solids.

[98] Mauro J.C., Loucks R.J., Sabyasachi S. (2010). Heat capacity, enthalpy fluctuations, and configurational entropy in broken ergodic systems. J. Chem. Phys..

[99] Patrascioiu A. (1987). The Ergodic Hypothesis: A Complicated Problem in Mathematics and Physics.

[100] Brush S.G. (2003). The Kinetic Theory of Gases.

[101] Palmer R.G. (1982). Broken ergodicity. Adv. Phys..

[102] Goldstein M. (2008). On the reality of residual entropies of glasses and disordered crystals. J. Chem. Phys..

[103] Goldstein M. (2011). On the reality of the residual entropy of glasses and disordered crystals: The entropy of mixing. J. Non-Cryst. Solids.

[104] Goldstein M. (2011). On the reality of the residual entropies of glasses and disordered crystals: Counting microstates, calculating fluctuations, and comparing averages. J. Chem. Phys..

[105] Johari G.P. (2011). Mechanical relaxation and the notion of time-dependent extent of ergodicity during the glass transition. Phys. Rev. E.

[106] Johari G.P. (2011). Specific heat relaxation-based critique of isothermal glass transition, zero residual entropy and time-average formalism for ergodicity loss. Thermochim. Acta.

[107] Richet P. (2009). Residual and configurational entropy: Quantitative checks through applications of Adam—Gibbs theory to the viscosity of silicate melts. J. Non-Cryst. Solids.

[108] Conradt R. (2009). On the entropy difference between the vitreous and the crystalline state. J. Non-Cryst. Solids.

[109] Fotheringham U., Baltes A., Müller R., Conradt R. (2009). The residual configurational entropy below the glass transition: Determination for two commercial optical glasses. J. Non-Cryst. Solids.

[110] Nemilov S.V. (2009). Zero-point entropy of glasses as physical reality. J. Non-Cryst. Solids.

[111] Johari G.P. (2010). On resolving the statistical and calorimetric entropies of glass and non-crystalline solids, and the residual entropy problem. Thermochim. Acta.

[112] Johari G.P., Khouri J. (2011). Entropy change on the cooling and heating paths between liquid and glass and the residual entropy. J. Chem. Phys..

[113] Johari G.P., Aji D.P.B., Gunawan L. (2011). Clausius limits on cooling and heating through the liquid—Glass range of three pharmaceuticals and one metal alloy-Annealing effects and residual entropy. Thermochim. Acta.

[114] Tombari E., Johari G.P. (2014). Change in entropy in thermal hysteresis of liquid-glass-liquid transition and consequences of violating the Clausius theorem. J. Chem. Phys..

[115] Schmelzer J.W.P., Gutzow I. (2009). Structural order-parameters, the Prigogine-Defay ratio, and the behavior of the entropy in vitrification. J. Non-Cryst. Solids.

[116] Tropin T.V., Schmelzer J.W.P., Schick C. (2011). On the dependence of the properties of glasses on cooling and heating rates. I. Entropy, entropy production, and glass transition temperature. J. Non-Cryst. Solids.

[117] Tropin T.V., Schmelzer J.W.P., Schick C. (2011). On the Dependence of the Properties of Glasses on Cooling and Heating Rates. II. Prigogine-Defay Ratio, Fictive Temperature, and Fictive Pressure. J. Non-Cryst. Solids.

[118] Jordanov N., Wondraczek L., Gutzow I. (2015). Thermodynamic properties of vitreous electrodes in a Ni/NiP glass-crystal Galvanic cell. J. Non-Cryst. Solids.

[119] Angell C.A. (2002). Liquid Fragility and the Glass Transition in Water and Aqueous Solutions. Chem. Rev..

[120] Mazurin O.V. (2007). Problems of Compatibility of the Values of Glass Transition Temperatures Published in the World Literature. Glass Phys. Chem..

[121] Cahn R.W. (1992). Missing atoms and melting. Nature.

[122] Stillinger F.H., Debenedetti P.G. (2013). Glass transition: Thermodynamics and kinetics. Annu. Rev. Condens. Matter Phys..

[123] Gupta P.K., Cassar D.R., Zanotto E.D. (2016). Role of dynamic heterogeneities in crystal nucleation kinetics in an oxide supercooled liquid. J. Chem. Phys..

[124] Kauzmann W. (1948). The Nature of the Glassy State and the Behavior of Liquids at Low Temperatures. Chem. Rev..

[125] Schmelzer J.W.P., Abyzov A.S., Fokin V.M. (2016). Thermodynamic Aspects of Pressure-Induced Crystallization: Kauzmann Pressure. Int. J. Appl. Glass Sci..

[126] Kauzmann W. (1993). Reminiscences from a life in protein physical chemistry. Protein Sci..

[127] Skripov V.P. (1974). Metastable Liquids.

[128] Schmelzer J.W.P., Abyzov A.S. (2011). On the theoretical description of nucleation in confined space. AIP Adv..

[129] Schmelzer J.W.P., Abyzov A.S., Baidakov V.G. (2017). Time of formation of the first supercritical nucleus, time-lag, and the steady-state nucleation rate. Int. J. Appl. Glass Sci..

[130] Angell C.A., MacFarlane D.R., Oguni M., Angell C.A., Goldstein M. (1986). The Kauzmann Paradox, Metastable Liquids, and Glasses: A Summary. Dynamic Aspects of Structural Change in Liquids and Glasses.

[131] Kalinina A.M., Filipovich V.N., Fokin V.M. (1980). Stationary and non-stationary crystal nucleation rate in a glass of 2Na_2_O·CaO·3SiO_2_ stoichiometric composition. J. Non-Cryst. Solids.

[132] Rouxel T. (2007). Elastic properties and short-to medium-range order in glasses. J. Am. Ceram. Soc..

[133] Schmelzer J.W.P., Abyzov A.S. (2018). Pressure-induced crystallization of liquids: Maxima of nucleation, growth, and overall crystallization rates. Int. J. Appl. Glass Sci..

[134] Schick C., Androsch R., Schmelzer J.W.P. (2017). Homogeneous crystal nucleation in polymers. J. Phys..

[135] Schmelzer J.W.P., Abyzov A.S. (2017). Crystallization of glass-forming melts: New answers to old questions. J. Non-Cryst. Solids.

[136] Zhuravlev E., Schmelzer J.W.P., Abyzov A.S., Fokin V.M., Androsch R., Schick C. (2015). Experimental test of Tammann’s nuclei development approach in crystallization of macromolecules. Cryst. Growth Des..

[137] Takada A., Conradt R., Richet P. (2015). Residual entropy and structural disorder in glass: A review of history and an attempt to resolve two apparently conflicting views. J. Non-Cryst. Solids.

[138] Simon F. (1937). On the range of stability of the fluid state. Trans. Faraday Soc..

[139] Bernal J.D. (1937). An attempt at a molecular theory of liquid structure. Trans. Faraday Soc..

[140] Frenkel Y.I. (1946). The Kinetic Theory of Liquids.

[141] Skripov V.P., Baidakov V.G. (1972). Absence of a spinodal in undercooled liquids. Teplofizika Vysokikh Temperatur.

[142] Skripov V.P., Faizullin M.Z. (2006). Crystal-Liquid-Gas Phase Transitions and Thermodynamic Similarity.

[143] Angell C.A. (2015). On the uncertain distinction between fast landscape exploration and second amorphous phase (ideal glass) interpretations of the ultrastable glass phenomenon. J. Non-Cryst.Solids.

[144] Stillinger F.H., Debenedetti P.G., Truskett T.M. (2001). The Kauzmann Paradox Revisited. J. Phys. Chem. B.

[145] Das S.P. (2011). Statistical Physics of Liquids at Freezing.

[146] Androsch R., Schick C., Schmelzer J.W.P. (2014). Sequence of enthalpy relaxation, homogeneous nucleation, and crystal growth in glassy Polyamide 6. Eur. Polym. J..

[147] Schmelzer J.W.P., Schick C. (2012). Dependence of Crystallization Processes of Glass-forming Melts on Prehistory: A Theoretical Approach to a Quantitative Treatment. Phys. Chem. Glasses B.

